# Learning from the community: iterative co-production of a programme to support the development of attention, regulation and thinking skills in toddlers at elevated likelihood of autism or ADHD

**DOI:** 10.1186/s40900-025-00674-7

**Published:** 2025-01-24

**Authors:** Alexandra Hendry, Victoria Hulks, Shona Murphy, Holly Radford, Sally Smith, Tony Charman, Sandra Mathers, Sinead Rhodes, Gaia Scerif

**Affiliations:** 1 Department of Experimental Psychology, University of Oxford, Oxford, UK; 2 History, Geography and Social Sciences Department, Edge Hill University, Oxford, UK; 3 Department of Psychology, University of Portsmouth, Oxford, UK; 4 Present Address: Peeple, Oxford, UK; 5 Present Address: Institute of Psychiatry, Psychology and Neuroscience, King’s College London, Oxford, UK; 6 Present Address: Department of Education, University of Oxford, Oxford, UK; 7 Present Address: Centre for Clinical Brain Sciences, University of Edinburgh, Oxford, UK

**Keywords:** Co-production, Patient and public involvement, Protocol development, Neurodiversity, Autism, ADHD, Executive functions

## Abstract

Programmes designed to support children with known, or increased likelihood of, autism or ADHD often focus on reducing behaviours central to a clinical diagnosis. However, supporting children to pursue their own goals and cope with everyday life through fostering executive function (EF) development, without enforcing neuro-normative assumptions, may be more acceptable to neurodivergent people, and more beneficial. The co-production process for this neurodiversity-affirming programme involved: Review of research priorities identified during published public-and-clinician consultations; iterative programme development through two pilot rounds with a general community sample; and consultation with stakeholders (parents with a connection to autism or ADHD, alongside early years specialists, psychologists and therapists) to check acceptability of the proposal, and refine the logic model and materials. The logic model for the resultant programme—Supporting Toddlers with a connection to autism or ADHD to develop strong Attention, Regulation and Thinking skills (START)—involves three mechanisms of change: The child has appropriate play-based opportunities to practise EF skills; Parenting behaviours linked to strong EFs are encouraged; Parents are empowered to improve environmental-fit for their child so that EF stressors are reduced.

**Supplementary Information:**

The online version contains supplementary material available at 10.1186/s40900-025-00674-7.

## Abstract (plain language summary)

Children showing many autistic traits, or who have a close family member on the autism spectrum or having ADHD, are more likely than average to struggle with attention, regulation and thinking skills. This may lead to difficulties with mental health and independent living in later life. We aimed to create a parent-toddler programme that would help children with a connection to autism or ADHD to thrive, without pressuring them to act in a certain way. To do this we first reviewed the results of previous studies and community consultations, and identified how we could build on an existing parent support programme structure to meet the project goals. Next, we tried out our ideas with 18 families. Then, we asked nine parents with a connection to autism or ADHD (because they are neurodivergent themselves, and/or raising a neurodivergent child), and five professionals to help us improve the materials further. The end result is a programme called START (Supporting Toddlers with a connection to autism or ADHD to develop strong Attention, Regulation and Thinking skills). This 12-week group-based parent-toddler programme aims to foster children’s development in three ways: (1) Giving children opportunities to practise their skills through play and everyday activities (2) Creating a welcoming, accessible and non-judgemental space for parents to hear about and share ideas (3) Helping parents to identify ways to help their child feel calm, safe and supported.

**Supplementary Information:**

The online version contains supplementary material available at 10.1186/s40900-025-00674-7.

## Background

In the UK, approximately 2.5% of children under seven years of age have a diagnosis of autism and/or Attention Deficit Hyperactivity Disorder (ADHD) [78]. Clinically, autism and ADHD are currently described as distinct conditions: Autism Spectrum Disorder (ASD) is characterised by difficulties in social communication and social interaction and by restricted, repetitive patterns of behaviour, interests, or activities, whereas ADHD is characterised by a persistent pattern of inattention and/or hyperactivity that interferes with functioning or development [3]. Yet, it is increasingly recognised that there are many overlapping aspects of experience in autism and ADHD, from hyperfocus [4, 36, 37] to heightened sensory profiles [80].

Indeed, autism and ADHD often co-occur within individuals [30], and tend to cluster in families; a child with an autistic first-degree relative is more likely than average to have ADHD, and vice versa (Ghirardi et al., 2018; 65). Both autism and ADHD have been linked to different (compared with neurotypical populations) profiles of executive functions—the higher-order cognitive skills which include inhibitory control, cognitive flexibility, and working memory, and which are important for planning, problem-solving and adapting to changing circumstances [18, 19, 77]. Older children and adults having autism or ADHD often show lower scores on measures indexing inhibitory control, working memory and planning, with cognitive flexibility difficulties being most linked to autism [23, 54, 71, 96]. Whilst differences are not inherently problematic, the executive function differences associated with autism and ADHD may cause difficulties with some aspects of day-to-day life and academic attainment [50, 61], and have been linked to poor mental health and lower quality of life [10, 22, 56, 84, 89].

Children with a family history of autism or ADHD are also more likely to experience some executive function related difficulties [9, 76, 83, 92]. Although executive function difficulties are less apparent on parent-reported measures for siblings of autistic children, compared with lab measures [62, 92], this may perhaps be attributable to ‘informant contrast effect’, where parents tend to underestimate the sibling's difficulties because they are implicitly compared to those of the older sibling.

Differences in executive functions emerge as early as toddlerhood (around 24 months of age) for children with a family history of autism or ADHD, with executive function difficulties (across multiple components) appearing particularly pronounced for those children already showing autistic traits [39, 68, 87]. Early attentional control influences later executive skills amongst children with and without a family history of autism [38, 42, 43]. Thus, supporting infants or toddlers at elevated likelihood of autism and ADHD to develop stronger attentional control (e.g. attention focus, attention shifting, selective attention) may also promote development of their executive functions. Acting as soon as attentional and executive skills exert influence over behaviour—in the second year of life [43]—may provide the greatest opportunities for positive effects to cascade down to other domains [1, 63, 70, 74].

Advocates of the neurodiversity paradigm argue that diversity of thought and behaviour should be embraced as a positive for society (whilst still acknowledging that some aspects of neurodivergence can be disabling at an individual level) and that supports should be intended to improve quality of life for the individual rather than to promote neurotypical behaviours [24, 51, 57]. Similarly, consultation with members of the autism and ADHD communities regarding priorities for applied health research have identified a preference for neurodiversity-affirming programmes of support which address factors that negatively impact on children’s well-being [8, 81]. Empowering children to pursue their own goals and cope with the day-to-day demands of life through supporting the development of executive function skills, without enforcing neuro-normative assumptions or targets, is, in principle, compatible with a neurodiversity perspective.

In co-producing a novel and neuro-diversity affirming programme supporting early executive functions, we drew from the extensive literature on the development and enrichment of executive functions for young children of the predominant neurotype (i.e. neurotypical children). Previous systematic reviews and meta-analyses of research on early executive function development have highlighted the importance of providing opportunities to practise EF skills across contexts and over time [5, 25, 82], and the need to adapt supports and skill-practise opportunities to individuals’ ability levels and interests [12, 25, 31]. Reviews also indicate that the parent–child relationship plays a key role in shaping early socio-emotional and cognitive development, and that reducing parent and child stress, and improving parent–child relationships may benefit executive function [11, 12, 55]. Particular parent behaviours have been associated with positive executive function development. These include providing autonomy support, sustained child routines, and stimulation that is developmentally appropriate, prompt, consistent, and contingent to the child’s cues and needs [12, 31, 43, 55, 75, 82, 91, 97]. For this reason, we identified that a parent-mediated programme would be most appropriate to meet the project aims.

In a systematic review and meta-analysis of parent-mediated interventions, Jeong et al. [49] concluded that interventions designed to promote responsive parenting behaviours were more effective at enhancing child cognitive development (broadly defined) compared to interventions that did not. More recently, Bennett et al. [7] evaluated a 10-week programme for parents of 12-to 36-month-olds comprising playgroup sessions supplemented with six home coaching visits focused around parenting skills such as providing routines, supporting children’s play, and following the child’s lead. They found that involvement in the programme was associated with higher child parent- and teacher-reported effortful control scores (pertaining to control of behaviour and attention and therefore related to executive function, but including some broader temperament-related items) at school age [7]. Such work indicates the importance of positive parent–child interactions and parenting strategies for supporting early cognitive and social-emotional development, consistent with the observational studies reported above. However, another meta-analysis has shown that positive parenting interventions had no significant benefits on executive functions specifically [73]. The authors conclude that early executive functioning development may be better targeted by programs that specifically target the cognitive control aspects of executive function, in addition to emotional regulation aspects [73]. It is, in part, this level of specificity that we aim to include in the current programme, and which is missing from existing intervention programmes.

Currently, there exist no intervention programmes for children under 3 years and their parents which combine positive parenting approaches with opportunities for children to practise executive functions specifically [27, 28, 49, 69, 73]. We therefore aimed to create a new programme to meet this need, and identified a suitable underpinning framework to follow in the Peep Learning Together Programme (LTP). The LTP, developed by Peeple [72], is an internationally-implemented evidence-based programme for parents and carers of infants through to pre-schoolers. Currently, the LTP targets development in communication, literacy, numeracy, health, physical, and social and emotional development via weekly group sessions. The LTP values and extends what parents already do to support their child's learning in everyday life, and takes a strengths-based approach to working with families, focusing on doing *with* rather than doing *to* parents. Each LTP session comprises songs, story-sharing, parent–child activities, guided discussions, and sharing of ideas to try at home, but the session structure and content can be adapted to suit the delivery context and content focus. Evaluations to date have indicated benefits for children’s literacy-related skills, parental self-acceptance, and parental confidence in learning and knowledge [64]. Since 2015, over 9000 practitioners, from all regions of the UK have been trained in the LTP, marking this out as a feasible, well-established model for intervention delivery. However, although the LTP aims to be inclusive to all families, it was not developed primarily with neurodiversity in mind and the LTP has not previously targeted executive functions directly.

To plan how executive function skills could be supported in the programme, we developed an outline content structure based on a hierarchical model of executive function development, whereby basic skills involving attentional control and impulse control act as foundational building blocks for more-complex executive functions [33, 43, 95]. In early child development executive functions operate in an integrative manner, such that targeting multiple executive function components is likely to be more effective than attempting to train skills in isolation [52]. Therefore the content structure was formulated as a pyramid, with later sessions involving activities that integrate skills from previous sessions alongside areas of previously-observed or potential strengths, in order to build self-esteem and self-efficacy. Such skills might include creative problem-solving (i.e. generativity); strengths in which have been observed at ages 2 and 3 years amongst children with a family history of ADHD [44].

Our next step was to refine this content structure, alongside the subsequent materials and underlying logic model. Co-production, as well as being morally preferable, is likely to maximise the uptake and effectiveness of executive function interventions [20, 25, 32]. Thus, the aim of this paper is to set out the co-production of a new neurodiversity-affirming parent-mediated programme aiming to support the development of executive functions in toddlers at elevated likelihood of autism or ADHD (by virtue of having a family history of autism or ADHD, or already showing elevated autistic traits).

## Methods

**Fig. 1:**
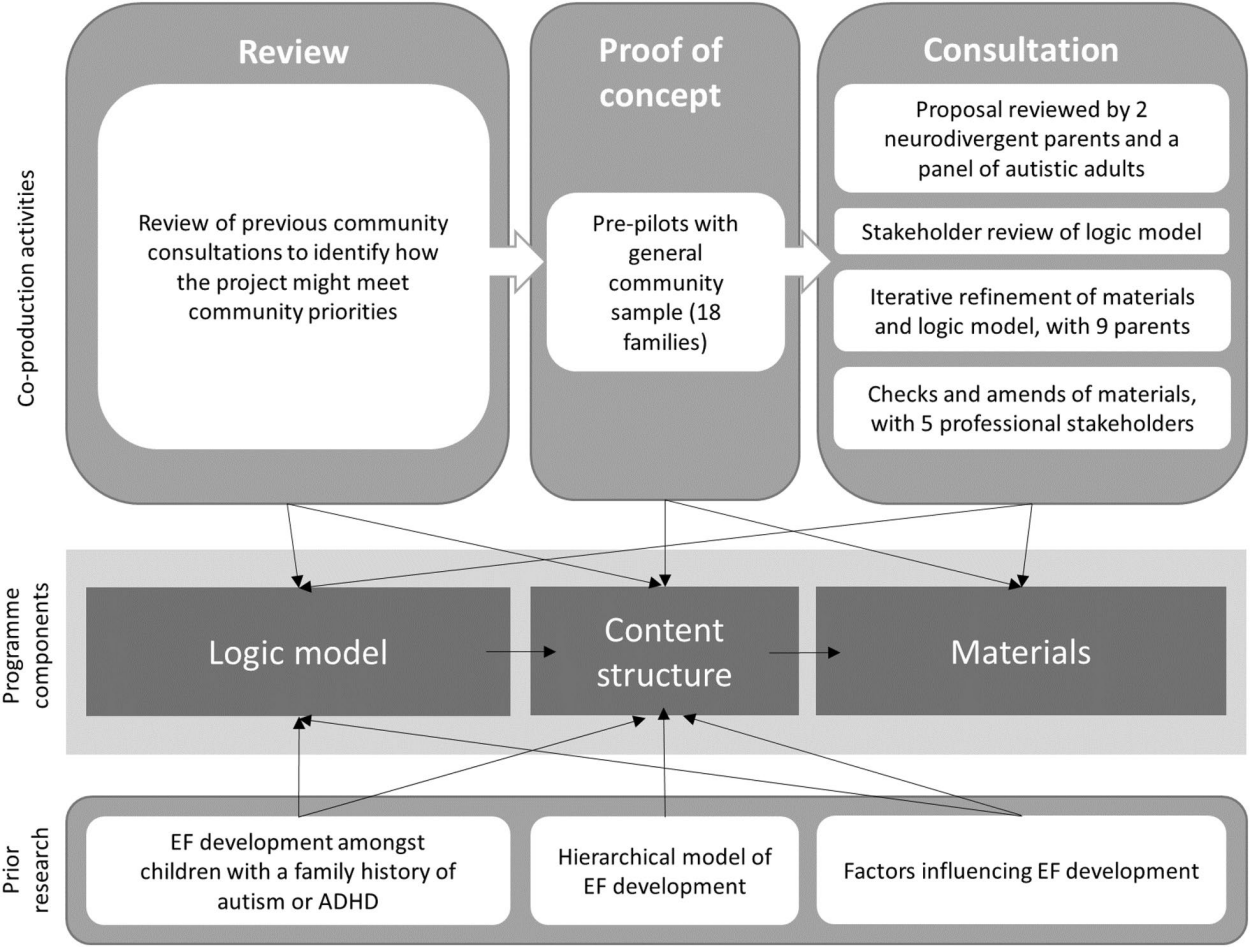
Development process for START

A 3-stage iterative approach was used to develop the logic model and programme materials, as shown in Fig. 1. This drew on guidance from the Medical Research Council (MRC) and the National Institute of Health and Care Research (NIHR) for developing complex interventions [85], examples of good practice in public health intervention development [60], principles of co-design [26, 79] and intervention mapping [6]. Results are reported in line with Guidance for reporting intervention development studies in health research [29]—see Appendix 1—and Guidance for Reporting Involvement of Patients and the Public [88]—see Appendix 2.

### Stage 1: review

Stage 1 involved reviewing what was already known about community priorities, and evidence-based ways to meet those priorities, within the identified problem-space of supporting the development of executive functions amongst children at elevated likelihood of autism or ADHD. James Lind Alliance (JLA) Priority Setting Partnerships (PSP) bring patient, carer and clinician groups together on an equal footing to identify and then jointly prioritise evidence uncertainties (i.e. questions which cannot be answered by existing research). PSPs must conform to a set of underpinning principles including transparency of process, balanced inclusion of patient, carer and clinician interests and perspectives, and exclusion of groups or organisations that have significant competing interests. They follow an established step-by-step process [2] which results in a list—often a Top 10—of jointly agreed research priorities. JLA PSPs thus offer a well-established route for identifying community research priorities. Therefore, as a first step the first author conducted a mapping exercise of the published PSP research priorities relevant to autism and ADHD. The aim of this step was firstly to establish whether a parent-mediated programme to support executive function development in toddlers at elevated likelihood of autism or ADHD can be considered broadly aligned to previously-identified community priorities for research, and secondly to identify opportunities to address some of these priorities within such a project (taking into account that not all of the priorities identified in the PSPs are compatible with the Neurodiversity paradigm adopted by this project).

Drawing on the findings from Stage 1, and theory drawn from the prior research summarised in the Background, we developed a draft programme logic model and preliminary programme curriculum.

### Stage 2: proof of concept

We next aimed to establish whether a parent-toddler programme focused on executive functions was acceptable to parents, and likely to increase engagement in parent–child executive function-related activities, and boost parent confidence in supporting their child’s executive functions. We also aimed to refine our ideas for activities and discussion to maximise engagement and potential impact. To achieve these aims, the draft programme was delivered in two rounds (of six hour-long sessions in each round) with a general community sample. Sessions were led by the programme developer alongside an experienced early years practitioner and one of two assistant facilitators (both parent/carers with an interest in child development). Participants were 18 families (9 per round) with children aged 7- to 19 months, recruited by the project partner (Peeple) from a socio-economically disadvantaged area of Oxford using their social media channels and local contacts; see Appendix 3a for participant details. The children involved had a deliberately wide age range as toddlers at elevated likelihood of autism/ADHD can be expected to show a wide range of developmental levels [17] and we wanted to identify activities that would be appropriate—or could be adapted to be appropriate—for children with varying cognitive, motor and language ability. This was important to accommodate individual differences in a group context, and to enable parents to extend the activity ideas as their child develops. The sessions were delivered in a community centre for round 1, and a room adjoining a nursery for round 2. Sessions followed the standard LTP structure: welcome song; topic discussion; parent–child activities; story-sharing and songs. Session content was planned in advance but adapted in the moment to meet the needs and interests of parents and toddlers, for example by repeating songs that toddlers found particularly engaging, and allowing discussions to be shaped by parental input. Activities were introduced by the practitioners, either by modelling play with one child initially, and then adding commentary on how the activities could be adapted or extended before sharing the relevant props or toys throughout the group; or by distributing the materials to each parent–child dyad initially, and then coaching the group step-by-step through the activity together.

Feedback was collected in real time through observations of parent and child responses to the activities and discussion prompts, reflections from the early years practitioner and assistant facilitator, and parent questionnaire after each session (see Appendix 3b). Using data collected via the parent questionnaire, a Wilcoxon signed ranks test of two related samples was used to compare usual practice frequency of involvement in parent–child executive function-related activities (‘How often do you already do the kinds of activities described in this session with your child?’) with anticipated post-session frequency of involvement in parent–child executive function-related activities (‘How likely are you to do the kinds of activities described in this session with your child over the next few weeks?’). Parent-reported confidence in the topic introduced in the session was summarised through descriptive statistics (mean, median and range) and additional comments gathered through free text was analysed using content analysis.

The programme was iteratively refined after each session based on in-session observations and written feedback. Activities were retained if: children were engaged; the activities were already at the appropriate level of challenge, or could easily be adapted; the activities were novel to the majority of families; and parents reported that they would try them at home. Discussion prompts were retained if they elicited discussion, and parents reported that they were confident about the topic after the session. Activities and discussion prompts that were less successful by these criteria were refined or replaced.

### Stage 3: consult

#### 3a Proposal

To ensure that the proposal was in line with current community priorities and perspectives, in November 2019–February 2020 feedback on the programme and its objectives were sought from two neurodivergent parents (one autistic, one with an autism-ADHD profile) approached by email by the first author (but previously unknown to her) after they were identified through social media as having an interest in supporting neurodivergent children to thrive. A broader perspective was then sought from a panel of six autistic adults who were consulted via the charity Autistica. Their written feedback was used to shape the funding proposal for the next stage of programme development and evaluation.

#### 3b Logic model

The draft programme logic model was presented for review by a panel of nine stakeholders via an online workshop held in June 2021. Five early years specialists with experience of developing, delivering or evaluating parent- and-toddler programmes, two developmental psychologists specialising in Executive Functions in the context of neurodiversity, and two neurodivergent researchers with a particular interest in parenting and positive supports for neurodivergent children, attended a two-hour workshop to review the draft programme logic model.

Stakeholders were identified through members of the project Steering Committee and personal contacts to represent a range of relevant personal and professional perspectives,. Stakeholders were asked to indicate how positively they felt about each component of the logic model, on a scale of 1 (very negative) to 5 (very positive), via an online discussion tool (Padlet). Low scores were prioritised for discussion, but additionally stakeholders could add comments to every section. Stakeholders were invited to comment on ways in which the logic model could be improved drawing on their professional and personal experience.

#### 3c Programme materials

The programme materials were iteratively refined in four online workshops held in July 2021 with a panel of eight parents. To provide checks that the core aims and underpinning logical model were appropriately represented in the programme materials, one panel member was involved in both Stage 2 and 3b. This kind of iterative approach is recommended to ensure better alignment of goals and outcomes [59]. To provide a range of perspectives, additional panel members were recruited via social media, emails to relevant charities and parent groups, and through word of mouth. To be eligible to participate, individuals needed to be a parent and either be autistic/have ADHD (diagnosed or self-identified), or have a child who is autistic/has ADHD (diagnosed or suspected). Panel members were invited to comment on ways in which the content could be improved (to improve inclusivity and alignment to a neurodiversity-affirming approach, or parent and child engagement more generally), and provide examples or tips from their own experience. Specifically, the panel were asked to input on the delivery approach, target child age and logic model, and the content of each proposed session. The detailed agenda and protocol for the workshops is presented in Appendix 4.

Following the workshops, the programme materials were updated to reflect the changes and additions suggested, and then sent out for review to six parents, five of whom had taken part in the initial workshops. Programme materials comprised: session plans (with comprehensive notes on discussion prompts, ideas for activities including adaptations and extensions and tips for delivery, song suggestions and a suggested book for story sharing; key ideas handouts; ‘to do at home’ activity handouts and details of the planned accompanying resources to be given to parents (valuing on average < £3 per family per session); and a manual detailing the programme principles, and guidance on ensuring sessions are accessible and inclusive.

The aims of these additional checks were to ensure that the recommendations discussed in the workshops were appropriately implemented, and to provide time for further in-depth reflection [59]. To complement this expertise-by-experience with insights from professionals with experience in supporting early cognitive development, the materials were sent for review to four external professional stakeholders (an early years practitioner, who is also a parent of an autistic child, an early years Special Educational Needs Speech and Language Therapist, a Music Therapist), and two members of a charity with the remit of supporting children’s early development through parent-mediated programmes.

#### Compensation

External professional stakeholders, parent panel members and the parents involved in the proposal consultation were compensated for their time and expertise at NIHR-Involve- suggested rates of £25 per hour. Time involvement in the review of the programme materials ranged from five hours (professional stakeholders and four parent panel members) to 10 h (two parent panel members), with the average time involvement of the nine parent panel members in the programme materials refinement phase being seven hours.

## Results

### Stage 1: Reviewing outputs from prior consultation exercises

At the time of review (October 2019), four PSPs relevant to autism/ADHD as specified in the PSP protocols themselves had been published by the JLA: Autism [21]; Childhood Disability [66]; Learning Difficulties (Scotland) [58]; and Neurodevelopmental Disorders (Canada) [53]. As detailed in Appendix 5, the proposal was potentially relevant for six of the top 10 research priorities identified in each of the Autism, Learning Difficulties (Scotland) and Neuro-developmental Disorders (Canada) PSPs. The proposal was identified as relevant to only two of the top 10 research priorities identified in the Childhood Disability PSP, but this was likely attributable to the broader remit of that PSP [66]. It was thus concluded that a programme to support children with a connection to autism or ADHD to develop strong executive functions was aligned with community priorities for research. The specific research priorities opportunities that might be addressed by the outline proposal are shown in Table 1.

**Table 1:** Summary of James Lind Alliance (JLA) Priority Setting Partnerships (PSP) priorities relevant to the proposal

PSP source	Priority	Relevance to programme^**a**^
Autism	Which interventions improve mental health or reduce mental health problems in autistic people? How should mental health interventions be adapted for the needs of autistic people?	EFs are implicated in mental health in typically-developing [86] and neurodivergent populations [56, 93, 94]: Secondary outcomes could include mental health
	Which interventions are effective in the development of communication/language skills in autism?	EFs are implicated in communication skills in typically-developing [14, 90] and autistic children [48]. Secondary outcomes could include communication skills
	Which interventions reduce anxiety in autistic people?	EFs are implicated in anxiety [56, 93, 94] amongst autistic people: Secondary outcomes could include anxiety
	Which environments/supports are most appropriate in terms of achieving the best education/ life/ social skills outcomes in autistic people?	Could address the role of environments/supports in supporting EF development
	How can parents and family members be supported/educated to care for and better understand an autistic relative?	Could incorporate neurodiversity-awareness into the programme, with parents as the programme recipient
	How can sensory processing in autism be better understood?	Could address the role of sensory processing in EF performance
Childhood Disability	Does the timing and intensity of therapies (e.g. physical, occupational and speech and language therapy, ‘early intervention’, providing information etc.) alter the effectiveness of therapies for infants and young children with neurodisability, including those without specific diagnosis? What is the appropriate age of onset / strategies / dosage / direction of therapy interventions?	Could evaluate the impact of age at enrollment and attendance levels on intervention outcomes (within a large-scale trial)
	To improve communication for children and young people with neurodisability: (a) what is the best way to select the most appropriate communication strategies? And (b) how to encourage staff/carers to use these strategies to enable communication?	EFs are implicated in communication skills in typically-developing [14, 90] and autistic children [48]. Secondary outcomes could include communication skills. Could include a range of strategies to facilitate communication
Learning Difficulties (Scotland)	What is the best educational and community environment for children and young people with learning difficulties?	Could address the role of environments/supports in supporting EF development
	Which early interventions are effective for children and young people with learning difficulties, at what ages and stages are they best introduced and what are the long-term outcomes?	Could evaluate the impact of age at enrollment on intervention outcomes (within a large-scale trial) and include long-term follow up
	How can parents, carers, brothers and sisters and extended families of children and young people with learning difficulties, be best supported to achieve their best quality of life before, during and after the diagnosis or identification in home, school and community contexts?	Could incorporate peer support into the programme, with parents as the programme recipient
	How can we best identify early features, symptoms and signs of learning difficulties amongst children, young people and their families/carers?	EFs are implicated in learning difficulties [50, 61]. Could collect measures of EF and broader developmental ability
	What is the best way to assess learning difficulties in children and young people?	As above
	Which strategies are effective in helping children and young people with learning difficulties live independent lives, including during times of transitions?	EFs are implicated in learning difficulties. Could include measures of adaptive function in long-term follow-up
Neuro-developmental Disorders (Canada)	What are the most effective treatment options/plans (e.g., timing, frequency, duration, type, intensity or dosage) for individuals with neurodevelopmental disorders for both short and long-term benefits?	Could evaluate the impact of age at enrollment and attendance levels on intervention outcomes (within a large-scale trial)
	Which child and family-centered interventions or approaches promote optimal individual and family functioning?	Could include a range of secondary outcomes relating to individual and family functioning
	Which interventions best help individuals with neurodevelopmental disorders develop emotional and behavioural regulation (including increasing impulse control and reducing compulsive behaviour)?	EFs are implicated in emotional and behavioural regulation. Secondary outcomes could include emotional and behavioural regulation
	Which are the most effective pharmacological and non-pharmacological treatments for aggressive and self-injurious behaviour in individuals with neurodevelopmental disorders?	EFs are implicated in aggressive behaviour in autism [56]. Secondary outcomes could include aggressive behaviour
	Which are the most effective pharmacological and non-pharmacological intervention(s) to reduce anxiety in individuals with neurodevelopmental disorders?	EFs are implicated in anxiety [56, 93, 94] amongst autistic people: Secondary outcomes could include anxiety
	Which interventions are most effective to help individuals with neurodevelopmental disorders improve their social skills and develop and maintain social relationships?	EFs are implicated in social skills in autistic children [48]. Secondary outcomes could include social skills

^a^As identified by the first author in terms of relevance to supporting the development of executive functions amongst children at elevated likelihood of autism or ADHD

EF: Executive functions

A draft programme logic model (see Appendix 6) and preliminary programme curriculum was developed, drawing on the findings from Stage 1, and designed to sit within the LTP framework: The lead author mapped out a hierarchical content structure based on models of executive function development in which attentional control and emotion regulation set the foundation for development of more-complex skills such as working memory and cognitive flexibility [43], then elaborated on this content structure to produce a detailed content map that included:

- Activity suggestions adapted from measures used in infant research to assess early executive functions [34, 35, 40, 41]. The rationale being that if a task can be used as an index of executive function, then adapting the task into a game provides an opportunity to practise the underlying executive function skill.
- Discussion guides and suggestions for ways to adapt the activity suggestions to real-world tasks (e.g. household chores, and day-to-day activities), and resources for parents to take home. The rationale here, is that skill practice will be more effective if embedded in everyday contexts and sustained over time.

This content map was reviewed by three early years specialists familiar with the LTP, who provided further ideas and links to existing LTP materials that could be repurposed, and suggestions for refinements to ensure appropriateness for families from socio-economically disadvantaged contexts.

### Stage 2: Proof of concept

Feedback questionnaire responses from participants who took part in pre-pilots of the draft programme are presented in Table 2. As this phase of the project was conducted as public engagement and not as a research activity, no personal data about participants was collected but a descriptive outline of the participants is presented in Appendix 3.

**Table 2:** Feedback on the draft programme, across participants (*n* = 18) and sessions (*n* = 6 in each of 2 rounds)

	*Pre-session activity engagement* How often do you already do the kinds of activities described in this session with your child? (Never = 1, Not very often = 2, Sometimes = 3, Often = 4)	*Post-session activity engagement* How likely are you to do the kinds of activities described in this session with your child over the next few weeks? (Not at all likely = 1, Not very likely = 2, Quite likely = 3, Very likely = 4)	How would you rate your confidence about this topic after the session? (Not at all confident = 1, Only a little confident = 2, Quite confident = 3, Very confident = 4)
Mean (SD)	3.26 (.94)	3.71 (.46)	3.33 (.55)
Median	4	4	3
Min, Max	1, 4	3, 4	2, 4

Parents reported being more likely to do executive function-related activities with their child having attended the sessions (*Z* = −2.909, *p* = 0.003). Overall, parents reported that they were confident about the target topic after each session.

Content analysis of additional written qualitative feedback at the end of the final session identified that parents appreciated the following aspects of the sessions (negative comments were explicitly elicited but none were made):

(i). Fun activities, songs and stories;

(ii). Clear explanations of the theory behind the games;

(iii). Supportive, relaxed atmosphere;

(iv). Appreciation for the take-home resources;

(v). Inspired to do games at home

(vi). Shared learning/ideas with family members

(vii). Gained insights about their individual child and how to support them.

Further opportunities to refine the draft programme based on observations of parent and child responses and practitioner feedback were also taken forward into the next stage of programme development. Some of the more-complex songs and activity suggestions were removed, details were added to session plans to address questions that parents raised in discussion, and an overview of how the different executive function components build on each other was added as a core visual aid. In response to some of the sessions feeling rushed or slightly overwhelming, the initial six-week programme was spread over 10 units. And, to accommodate the observation that even engaged parents were not able to attend every week, two recap or consolidation sessions were added (at weeks 6 and 12).

### Stage 3a: Consultation within community stakeholders to check acceptability of the programme and evaluation proposal

The results of a community review of the programme and its objectives identified many positive aspects and support for the basic premise. Areas of strengths identified by reviewers included: the potential for improving outcomes for all involved; that executive functioning is something that most autistic people would like to improve; and the efforts made to increase accessibility of the material. Although reviewers praised the sensitive and positive approach towards neurodiversity taken within the proposal, some suggestions for further improvements were made regarding language and further increasing the accessibility of the programme. Specific recommendations that were used to refine the proposal and subsequent programme included using identity-first language by default when discussing autistic people, using audio rather than video when recording sessions for fidelity-checking purposes, giving clear directions (with pictures) to the session venues and meeting parents at the door of the building rather than expecting them to get to the room on their own, and including self-identification of autism/ADHD as an acceptable criterion for inclusion because there are many barriers to diagnosis.

These suggestions were incorporated into the revised proposal as further detailed in Appendix 7.

### Stage 3b: Stakeholder review of the programme logic model

**Fig. 2:**
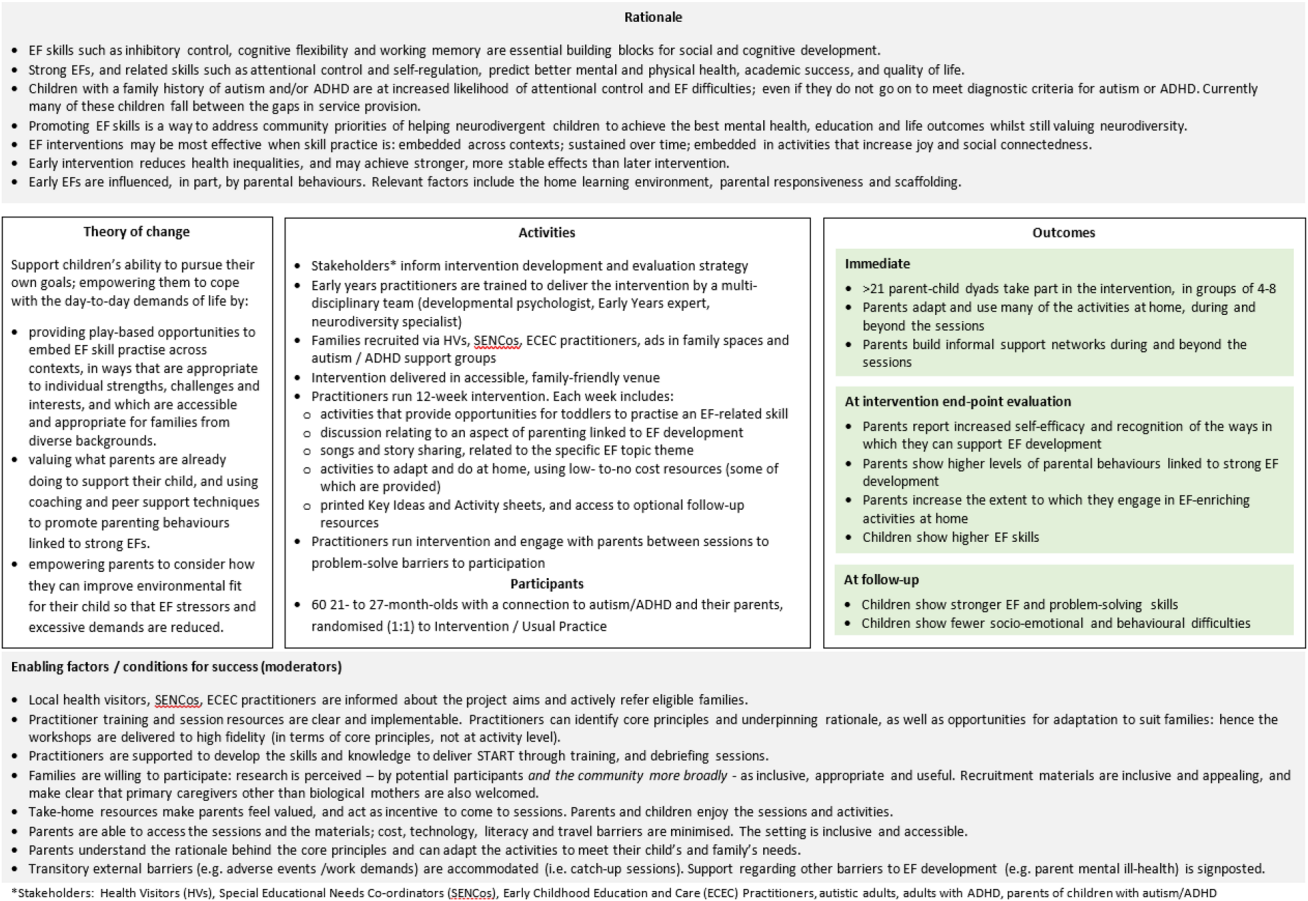
Logic model for the START programme

As shown in Appendix 8, each component of the logic model was endorsed by the Stakeholder group (mean average ratings for each topic were between 4 and 5 out of 5), but improvements and considerations were suggested for each component. This feedback was used to refine the logic model presented in Fig. 2 and, where appropriate, the programme materials themselves. During these discussions, members of the stakeholder group raised a concern that existing research measures relating to parenting tend to be developed by and with neurotypical populations and may not be appropriate for contexts where the parent, child or both is neurodivergent (see Appendix 8). They highlighted that using such measures to evaluate the impact of the START programme (specifically regarding changes in parental self-efficacy or in parental behaviours linked to strong executive function development, such as responsivity) might lead to invalid conclusions and perpetuate harmful stereotypes about neurodivergent parents. These concerns led to the initiation of a project to develop neurodiversity-affirming measures of parental self-efficacy [47] and parent responsivity [15, 16].

### Stage 3c: Co-refinement of the programme

Characteristics of the panel members involved in the workshops to review and refine the programme are presented in Table 3. Parents with children older than three years were asked to reflect on their experiences when their child was three years or younger. Parents attended between one and three workshops each (mean 2.36), with all panel members attending either Workshop 1a or 1b, followed by up to two further workshops from Workshops 2 and 3, depending on availability.

**Table 3:** Panel member profile (n = 9)

	Parent identifies* as being/having		Parent has a child who is identified* as being/having		Child(ren) age range					No. workshops attended	Reviewed revised materials
	Autistic	ADHD	Autistic	ADHD	< 3	3–6	7–10	11–14	> 14		
1	1	1	1	1	0	1	0	0	0	3	1
2	0	0	1	0	1	0	1	0	0	2	0
3	0	0	0	1	0	0	0	1	0	2	0
4	1	1	1	0	1	0	0	0	0	3	1
5	1	1	1	1	0	0	1	0	1	3	1
6	0	0	1	0	0	1	1	0	0	3	0
7	1	0	0	0	1	0	0	0	0	1	1
8	1	1	1	1	0	0	0	1	1	2	1
9	1	1	1	1	0	0	1	1	1	0	1

*Either by virtue of clinical diagnosis or self-identification

#### Delivery approach, target child age and logic model

Opinion was initially mixed on whether the sessions should be delivered online or face to face (with three versus five votes respectively), but by the end of Workshop 1 (having reviewed some of the materials), the panel were unanimously in favour of face-to-face delivery. This was primarily due to the advantages afforded by in-person groups for supporting the development of supportive peer networks for parents, and child friendships.

Panellists endorsed the programme name: Supporting Toddlers with a connection to autism or ADHD to develop strong Attention, Regulation and Thinking Skills (START).

Based on the panel input, the target child starting age was raised to 21- to 27-months, so that most children attending would be likely to be able to engage in the proposed activities at an appropriate level of challenge (taking into account likely diversity in language skills, motor control and executive functions within the target population).

The Theory of Change was refined to include the component *empowers parents to consider how they can improve environmental fit for their child so that executive function stressors and excessive demands are reduced*—see Fig. 2. Accordingly, session plans for each topic were revised to include prompts for parents to consider what supports and accommodations might be helpful to reduce executive function stressors (e.g. sensory overwhelm, anxiety [45]), and how they might adapt games and day-to-day activities to ensure the level of executive challenge is appropriate for their child.

#### Evaluation strategy

The detailed evaluation strategy was reviewed by a subset of panel members and the programme steering committee, as described in [TRIAL PROTOCOL] and was not a focus of the programme co-refinement. However, during the parent panel workshops it was noted by multiple parents that most group parent-toddler sessions are not experienced as inclusive or accessible (e.g. due to perceived expectations to adhere to neurotypical social norms, noisy and/or unpredictable sessions, and neuro-normative developmental expectations for children), and that therefore the proposed plan for the Feasibility Trial to assign families randomised to an active control arm to attend a standard parent-toddler programme was not acceptable. Consequently, the protocol was revised (with approval from the project Steering Committee and Funder) to include only Usual Practice as the control arm.

#### Session content

**Fig. 3:**
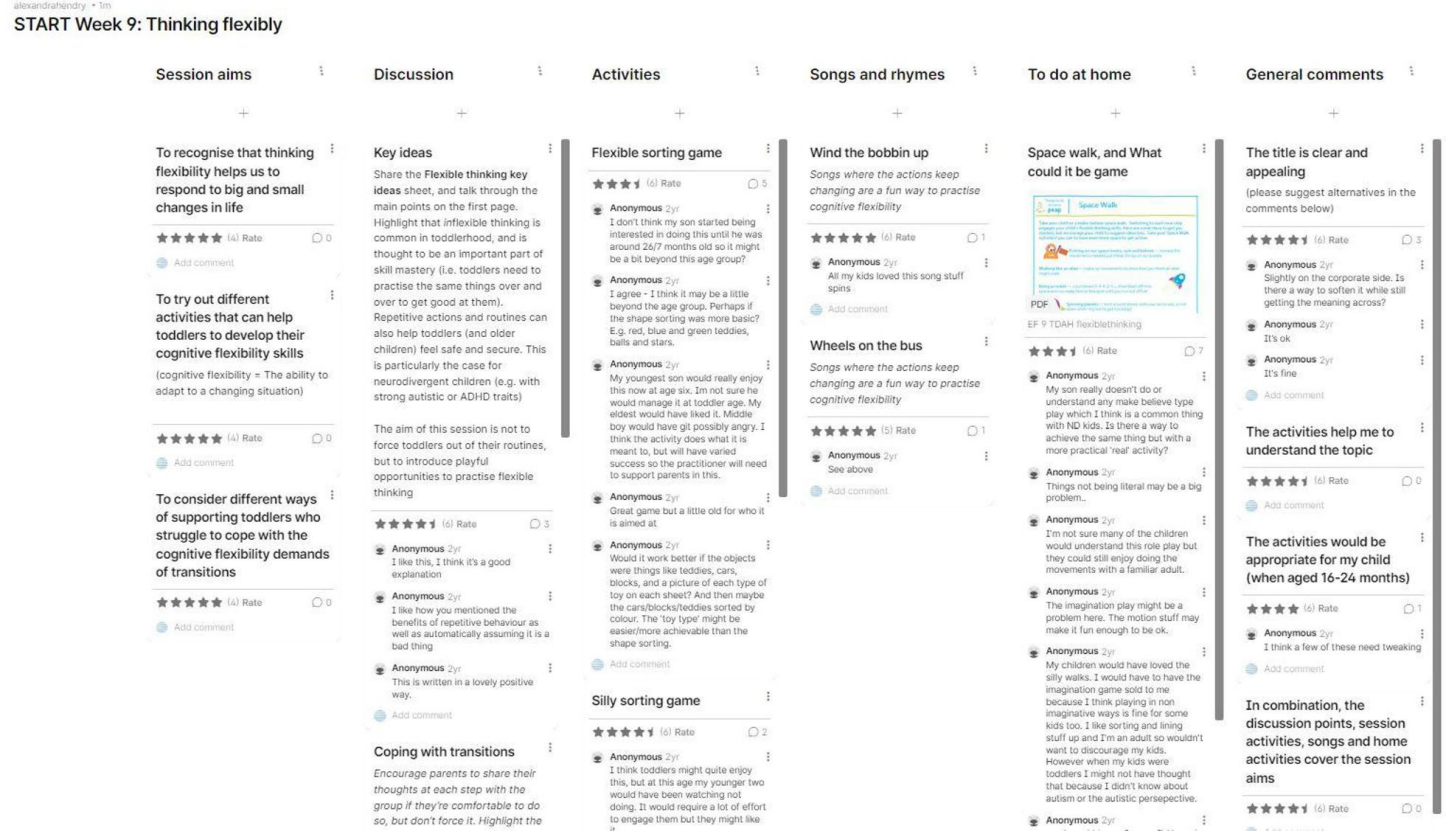
A completed Padlet discussion for Session 9

Each of the proposed topics was viewed positively (mean average ratings for each topic were between 4 and 5 out of 5), but improvements were suggested for the detailed content in each sub-section. Some activities were identified as challenging even with the higher child starting age but still suitable for inclusion if framed correctly (i.e. to help parents understand the kinds of skills their child could work towards, and as ideas for activities to try after the direct delivery phase had finished) as this provided a way of extending the long-term impact of the programme. An example of the in-depth review of the session materials via Padlet is presented in Fig. 3. The activity ideas, handouts and discussion guides were updated in line with the suggestions made by the panel members. Additionally, a new component—Parent insights—was added to the session plans, featuring quotes and examples from the panel members. This had the function of explicitly celebrating parental expertise and neurodivergent experiences throughout the programme.

### Stage 3d: Further revisions

The revised materials were sent out for review to the six panel members who indicated they had the capacity for further involvement, and five professionals with experience in supporting early cognitive development. Panel members were not asked to disclose ethnicity at the time of recruitment but it was observed during the workshops that all members were perceived as White. Therefore, to go some way towards mitigating against cultural blind-spots, additional review was sought from a neurodivergent parent from a minoritized background with a particular interest and professional background in intersectionality of race and neurodivergence, who was asked to provide additional comment on the suitability of the materials from that perspective.

As shown in Appendix 9, the response to the revised materials was overwhelmingly positive. Nevertheless, the reviewers did make some detailed suggestions for improvements to improve inclusivity, including some suggestions for alternative books to use during the story sharing part of the session, and adaptations to suggest to parents for some of the activities if their child was not motivated or appropriately challenged by the core activity, all of which were incorporated into the materials before progression to Stage 3.

## Discussion

### Programme summary and theory of change

Using a three-stage, iterative approach to embed community priorities and perspectives in every stage of development, we have created a new programme—Supporting Toddlers with a connection to autism or ADHD to develop strong Attention, Regulation and Thinking Skills (START). This programme aims to support children at elevated likelihood of autism or ADHD (by virtue of showing many autistic traits, or having a family history of autism or ADHD) to thrive by promoting their executive function development. The theory of change is that parents who take part in the programme will report increased self-efficacy and recognition of the ways in which they can support their child’s executive function development, show higher levels of parental behaviours linked to strong executive function development (such as scaffolding and responsivity), and increase the extent to which they engage in executive function-enriching activities at home; both during the delivery phase of the project, and beyond. In turn, this is intended to lead to children showing stronger executive function and problem-solving skills and fewer socio-emotional and behavioural difficulties at follow-up. To achieve this, programme materials comprise: 10 unique session plans (featuring discussion prompts, parent insights, activities with suggested adaptations and extensions, song suggestions, and a book suggestion for story sharing); key ideas handouts; ‘to do at home’ activity handouts with accompanying physical resources; and a manual detailing the programme principles alongside guidance on ensuring sessions are accessible and inclusive.

To develop the programme we first reviewed the results of four extensive consultation exercises to ensure that our aim (to promote executive function development amongst children having a connection to autism or ADHD via a parent-toddler programme) was aligned with previously-identified community priorities for research. We then tested the intervention concept (a semi-structured parent-toddler programme focused on activities and parenting approaches likely to promote attentional control and executive functions) with a general community sample. This allowed us to check that the approach was broadly feasible, acceptable and likely to effect change, and also take account of children’s perspectives by iteratively developing the programme in response to their—and their parent’s—reactions during the sessions. We next took a step back to consult with autism-ADHD community members to double-check the acceptability of the programme and evaluation proposal before refining the logic model with input from a broader range of stakeholders (parents, researchers and early years specialists). We then iteratively refined the detailed programme materials in collaboration with nine parents who were either neurodivergent themselves, raising a neurodivergent child, or both. This multi-step approach ensured value for money and efficient use of stakeholders’ time by building on existing consultation exercises, and allowed us to draw on a breadth of views and expertise whilst also gaining in-depth feedback when needed.

### Co-design successes and lessons learned

Fair compensation for advisory group members, flexible opportunities to input, and clear communication of input parameters at each point, allowed capture of a diverse range of experiences and perspectives whilst maintaining project momentum and a coherent strategy. Following the Guided checklist [29] supported transparent reporting through the development process. These strengths were made possible due to the funder’s support in providing sufficient budget and time to undertake co-development.

As is perhaps inevitable in any community consultation exercise, stakeholders did not always initially make the same recommendations. However, through open, respectful discussion of pros and cons of different recommendations in the light of the specific goals of the project, consensus was generally reached. For example, opinion was initially mixed on the question of whether the sessions should be delivered online or face-to-face but was unanimously in favour of face-to-face delivery once the sample materials had been reviewed. Where a one-size-fits-all solution was not appropriate, advisory group members were happy to endorse principle-based decision making. One example of this was the terminology used to describe the target population; whilst most stakeholders preferred identity-first language for autism (i.e. ‘autistic person’) they were aware that not all parents would share this view, and also that there is not yet a well-established identity-first formulation for ADHD. Therefore, the recommendation was to use identity-first language by default when discussing autistic people, but to include a position statement on the project website and in the programme manual endorsing the importance of respecting individuals’ preferences. This approach is also in line with recommendations for avoiding ableist language, drawn from other consultations with autistic adults as described by Bottema-Beutel et al. [13].

Several lessons were learned during the co-design process which may be useful for other researchers. The first was about the value of explicitly encouraging constructive criticism. Initial consultation during Stage 3a (conducted remotely and mostly over email) was very positive but did not lead to substantive changes to the project. To ensure that the community engagement was not tokenistic, for stages 3b onwards consultation was primarily via online meetings, at the start of which participants were explicitly asked to focus on areas for potential improvement or change. This set a positive collaborative tone for subsequent discussions and provided the conditions to enable the parent panel to recommend the addition of a third component to the Theory of Change; to empower parents to consider how they can improve environmental fit for their child so that executive function stressors and excessive demands are reduced. This shift from not just aiming to support toddlers to develop stronger executive function skills, but also to accommodate their current needs and abilities, was one of the most fundamental changes to the project, leading to several new session discussion prompts, adaptations to the key ideas sheets, and additional ideas for how to adapt activities to provide an appropriate but not overwhelming level of challenge.

Alongside this, we learned the value of being transparent about the constraints of the project in terms of deadlines and budget limits for decisions, delivery and evaluation. Advisory group members responded well to these constraints, mostly targeting their feedback and suggestions to what was possible within *this* project. When discussion strayed outside these parameters—for example with comments about the need for neurodiversity-affirming training in supporting executive functions for early years practitioners and Health Visitors, and for more appropriate measures of parenting (see below for further discussion)—the research team reiterated the project constraints and checked that advisory group members were content for these to be documented as suggestions to inform future, related projects.

A related lesson learned was not to immediately drop components of the programme or the evaluation in response to one or two individual comments, but also to be open to change. For example, some specific activities were flagged by a few parents as being unappealing to them/their child, but were seen positively by others. Rather than remove them from the session plans, we added suggestions for alternatives, along with contextual information for practitioners about why this might be needed (e.g. because the activity relied on imaginary play, which some autistic adults and children find difficult or unappealing). In another example, several neurodivergent members of the stakeholder group raised concerns about evaluating parental self-efficacy and parental responsivity due to the risk that using currently available measures might lead to invalid conclusions and perpetuate harmful stereotypes about neurodivergent parents. Our initial response was to therefore to drop this aspect from the evaluation plan. However, other members of the stakeholder group flagged that this would undermine our ability to test two key elements of the programme theory of change. Through further consultation we identified that the best way forward would in fact be to initiate a project to develop neurodiversity-affirming measures of parental self-efficacy and parent responsivity; in this way we have been able to address community concerns whilst retaining a robust evaluation approach.

### Project limitations

A limitation of the co-refinement process is the lack of direct input (although some parent contributors did indirectly represent their perspective) from minimally-verbal or intellectually-disabled members of the neurodivergent community, who remain under-represented in coproduction work. Further, as much of this work was conducted as a consultation exercise and not within the ethically-approved research activity, we did not collect data on the demographic characteristics of Advisory Group members. Consequently, we were unable to report in detail on their representativeness in terms of ethnic or socio-economic characteristics, but are aware that we likely have only limited input from individuals from marginalised groups during the development process. Efforts to redress this imbalance have been made during the next phases of the project, by recruiting pilot and feasibility trial participants from socio-economically and ethnically-diverse local populations, with varying family structures, and paying particular attention to indicators that the programme may not be appropriate for particular demographic groups.

We took several steps to maximise diversity of perspectives of the Advisory Group members; such as by approaching people not previously known to the research team to avoid an echo chamber [32], and by engaging with an independent review panel convened by an autism charity. Individuals who have the capacity or confidence to engage with co-production of research may not fully represent the priorities, perspectives and barriers faced by those we seek to support directly with the programme. However, these individuals are often leaders in the community, have a good understanding of the research process and its limitations, and can represent a wider range of interests than just their own, making their contributions very insightful [67]. Nevertheless, our approach of identifying panel members via social media, emails to relevant charities and parent groups, and word of mouth may have introduced some sampling bias. Therefore, the next stage of development and evaluation is a feasibility and acceptability pilot of the programme specifically with a diverse group of families having a connection to autism or ADHD [47], followed by a feasibility RCT of the START programme and evaluation strategy (using an evaluation protocol also co-developed with the community [46].

## Conclusion

Combining community consultation with evidenced-based theory has facilitated the development of a novel programme which addresses community needs and priorities by supporting executive function development amongst toddlers who have a connection to autism or ADHD. We have been able to maximise the quality and potential effectiveness of the programme through incorporating the extensive expertise by experience of parents who are neurodivergent themselves and/or have a child who is autistic or has ADHD. In summary, the co-production impacted the programme in the following ways: (i) influenced the approach to what language would be used (ii) added a component to the theory of change of the intervention, (iii) determined the delivery approach as in person opposed to online (iv) influenced the content of the intervention session plans and handouts (v) influenced the decision to develop two neurodiversity-affirming measures of parenting to be used to evaluate potential mechanisms of change. Key features of the resultant programme are that it combines play-based, individualised cognitive training for the child, with coaching and peer-support based psycho-education. This aims to promote parenting behaviours linked to strong executive functions, and empower parents to consider how they can improve environmental fit for their child so that executive function stressors and excessive demands are reduced. The programme has been developed within a neurodiversity-affirming perspective which values and recognises what parents are already doing to support their child, meets the child where they are, and encourages them as individuals—with unique strengths, challenges and interests—to thrive.

## Supplementary Information

Additional file 1.

Additional file 2.

## Data Availability

Data to support this article are available from the author on reasonable request. No datasets were generated or analysed during the current study.

## References

[1] Ahmed SF, Kuhfeld M, Watts TW, Davis-Kean PE, Vandell DL. Preschool executive function and adult outcomes: a developmental cascade model. Dev Psychol. 2021;57(12):2234. 34928671 10.1037/dev0001270

[2] Alliance TJL. The James Lind Alliance Guidebook (Version 10);2021

[3] APA. Diagnostic and statistical manual of mental disorders (5th ed.). APA; 2013. <Go to WoS>://MEDLINE:24670961

[4] Ashinoff BK, Abu-Akel A. Hyperfocus: the forgotten frontier of attention. Psychol Res. 2021;85(1):1–19. 10.1007/s00426-019-01245-8. 31541305 10.1007/s00426-019-01245-8 PMC7851038

[5] Baron A, Evangelou M, Malmberg L-E, Melendez-Torres GJ. The Tools of the Mind curriculum for improving self-regulation in early childhood: a sytematic review. Campbell Syst Rev. 2017;13(1):1–77. 10.4073/csr.2017.10.

[6] Bartholomew LK, Parcel GS, Kok G. Intervention mapping: a process for developing theory and evidence-based health education programs. Health Educ Behav. 1998;25(5):545–63. 9768376 10.1177/109019819802500502

[7] Bennett C, Westrupp EM, Bennetts SK, Love J, Hackworth NJ, Berthelsen D, Nicholson JM. An early parenting intervention focused on enriched parent–child interactions improves effortful control in the early years of school. Child Dev. 2024. 10.1111/cdev.14166. 39359116 10.1111/cdev.14166 PMC11693840

[8] Bent CA, Aulich A, Fidock E, Constantine C, Gurba AN, Dwyer P, Harrington L, Smith J, Gore KE, Rabba AS, Green CC, Hudry K. Autistic and autism communities’ perspectives on providing supports to infants and their families very early in life. Stockholm: INSAR; 2023.

[9] Bidwell LC, Willcutt EG, DeFries JC, Pennington BF. Testing for neuropsychological endophenotypes in siblings discordant for attention-deficit/hyperactivity disorder. Biol Psychiatry. 2007;62(9):991–8. 10.1016/j.biopsych.2007.04.003. 17585884 10.1016/j.biopsych.2007.04.003 PMC2687149

[10] Bishop-Fitzpatrick L, Hong J, Smith LE, Makuch RA, Greenberg JS, Mailick MR. Characterizing objective quality of life and normative outcomes in adults with autism spectrum disorder: an exploratory latent class analysis. J Autism Dev Disord. 2016;46(8):2707–19. 10.1007/s10803-016-2816-3. 27207091 10.1007/s10803-016-2816-3 PMC5039043

[11] Blair C. Developmental science and executive function. Curr Dir Psychol Sci. 2016;25(1):3–7. 10.1177/0963721415622634. 26985139 10.1177/0963721415622634 PMC4789148

[12] Blair C, Raver CC. Poverty, stress, and brain development: new directions for prevention and intervention. Acad Pediatr. 2016;16(3):S30–6. 27044699 10.1016/j.acap.2016.01.010 PMC5765853

[13] Bottema-Beutel K, Kapp SK, Lester JN, Sasson NJ, Hand BN. Avoiding ableist language: suggestions for autism researchers. Autism Adulthood. 2021; 10.1089/aut.2020.0014 PMC8992888 36601265

[14] Bruce M, Bell MA. Vocabulary and executive functioning: a scoping review of the unidirectional and bidirectional associations across early childhood. Hum Dev. 2022;66(3):167–87. 10.1159/000524964. 36164662 10.1159/000524964 PMC9501766

[15] Castle V, Hulks V, Mathers S, Hendry A. Observational measures of parent-child interaction used with neurodivergent parents or infants: a systematic review;2024. https://osf.io/meq82/

[16] Castle V, Taylor J, Hulks V, Gupta M, Maccallum C, Croke C, Mathers S, Hendry A. The inclusive assessment of parent child interaction (IAPCI): a neurodiversity-affirming measure of responsive parenting; 2024. https://osf.io/c6rj5/

[17] Charman T, Pasco G, Hendry A, Bazelmans T, Narvekar N, Goodwin A, Halkola H, Agyapong M, Holman R, Ali JB. Three year outcomes in infants with a family history of autism and/or attention deficit hyperactivity disorder. JCPP Adv. 2023;3(4): e12189. 38054052 10.1002/jcv2.12189 PMC10694531

[18] Christoforou M, Jones EJ, White P, Charman T. Executive function profiles of preschool children with autism spectrum disorder and attention-deficit/hyperactivity disorder: a systematic review. JCPP Adv. 2023;3(1): e12123. 37431322 10.1002/jcv2.12123 PMC10241451

[19] Craig F, Margari F, Legrottaglie AR, Palumbi R, Giambattista C, Margari L. A review of executive function deficits in autism spectrum disorder and attention-deficit/hyperactivity disorder. J Neuropsychiatric Dis Treat. 2016;12:1191–202. 10.2147/NDT.S104620 PMC4869784 27274255

[20] Crocker JC, Ricci-Cabello I, Parker A, Hirst JA, Chant A, Petit-Zeman S, Evans D, Rees S. Impact of patient and public involvement on enrolment and retention in clinical trials: systematic review and meta-analysis. Bmj. 2018;363. 10.1136/bmj.k4738 PMC6259046 30487232

[21] Cusack J, Sterry R. Your Questions: Shaping Future Autism Research; 2016

[22] de Vries M, Geurts HM. Influence of autism traits and executive functioning on quality of life in children with an autism spectrum disorder. J Autism Dev Disord. 2015;45(9):2734–43. 10.1007/s10803-015-2438-1. 25835211 10.1007/s10803-015-2438-1 PMC4553152

[23] Demetriou EA, Lampit A, Quintana DS, Naismith SL, Song YJC, Pye JE, Hickie I, Guastella AJ. Autism spectrum disorders: a meta-analysis of executive function. Mol Psychiatry. 2017. 10.1038/mp.2017.75. 28439105 10.1038/mp.2017.75 PMC5984099

[24] Den Houting J. Neurodiversity: An insider’s perspective, vol. 23. London: Sage; 2019. p. 271–3. 10.1177/1362361318820762 30556743

[25] Diamond A, Ling DS. Conclusions about interventions, programs, and approaches for improving executive functions that appear justified and those that, despite much hype, do not. Dev Cogn Neurosci. 2016;18:34–48. 10.1016/j.dcn.2015.11.005. 26749076 10.1016/j.dcn.2015.11.005 PMC5108631

[26] Donald S, Gaigg S, Edwards N, Remington A, Henry L. Co-designing research methods improves accessbility and inclusivity for under-represented groups, especially autistic people with complex support needs. INSAR, Stockholm; 2023

[27] Duncan AF. Interventions for executive function in high-risk infants and toddlers. Clin Perinatol. 2023;50(1):103–19. 10.1016/j.clp.2022.10.003. 36868701 10.1016/j.clp.2022.10.003

[28] Duncan AF, Gerner GJ, Neel ML, Burton VJ, Byrne R, Warschausky S. Interventions to improve executive functions in children aged 3 years and under: a systematic review. Child Care Health Dev. 2024;50(4): e13298. 10.1111/cch.13298. 38958229 10.1111/cch.13298

[29] Duncan E, O’Cathain A, Rousseau N, Croot L, Sworn K, Turner KM, Yardley L, Hoddinott P. Guidance for reporting intervention development studies in health research (GUIDED): an evidence-based consensus study. BMJ Open. 2020;10(4): e033516. 32273313 10.1136/bmjopen-2019-033516 PMC7245409

[30] Eaton C, Roarty K, Doval N, Shetty S, Goodall K, Rhodes SM. The prevalence of attention deficit/hyperactivity disorder symptoms in children and adolescents with autism spectrum disorder without intellectual disability: a systematic review. J Atten Disord. 2023;27(12):1360–76. 10.1177/10870547231177466. 37287320 10.1177/10870547231177466 PMC10498659

[31] Fay-Stammbach T, Hawes DJ, Meredith P. Parenting influences on executive function in early childhood: a review. Child Dev Perspect. 2014;8(4):258–64. 10.1111/cdep.12095.

[32] Fletcher-Watson S, Brook K, Hallett S, Murray F, Crompton CJ. Inclusive practices for neurodevelopmental research. Curr. Dev. Disorders Rep. 2021;1–10.

[33] Garon N, Bryson SE, Smith IM. Executive function in preschoolers: a review using an integrative framework. Psychol Bull. 2008;134(1):31–60. 10.1037/0033-2909.134.1.31. 18193994 10.1037/0033-2909.134.1.31

[34] Garon N, Smith IM, Bryson SE. A novel executive function battery for preschoolers: sensitivity to age differences. Child Neuropsychol. 2014;20(6):713–36. 10.1080/09297049.2013.857650. 24295496 10.1080/09297049.2013.857650

[35] Goldman DZ, Shapiro EG, Nelson CA. Measurement of vigilance in 2-year-old children. Dev Neuropsychol. 2004;25(3):227–50. 10.1207/s15326942dn2503_1. 15147998 10.1207/s15326942dn2503_1

[36] Grant A, Kara H. Considering the Autistic advantage in qualitative research: the strengths of Autistic researchers. Contemp Soc Sci. 2021;16(5):589–603. 10.1080/21582041.2021.1998589.

[37] Grotewiel MM, Crenshaw ME, Dorsey A, Street E. Experiences of hyperfocus and flow in college students with and without attention deficit hyperactivity disorder (ADHD). Curr Psychol. 2022. 10.1007/s12144-021-02539-0.

[38] Hendry A. Investigating the Early Development of Control of Attention and Executive Function in Children at Risk for Autism Spectrum Disorder. King's College London;2018.

[39] Hendry A, Bedford R, Agyapong M, Begum Ali J, Bazelmans T, Ersoy M, Goodwin A, Mason L, Narvekar N, Pasco G, Johnson MH, Jones EJH, Charman T, Team TS. Simple Executive Function as an endophenotype of autism-ADHD, and differing associations between simple versus complex Executive Functions and autism/ADHD traits. OSF. 2024; 10.1038/s41598-025-87863-2 PMC11811128 39929907

[40] Hendry A, Greenhalgh I, Bailey R, Fiske A, Dvergsdal H, Holmboe K. Development of directed global inhibition, competitive inhibition and behavioural inhibition during the transition between infancy and toddlerhood. Dev Sci. 2022;25(5): e13193. 34811852 10.1111/desc.13193 PMC11475536

[41] Hendry A, Holmboe K. The early executive functions questionnaire: validation of a new parent-report measure for 9- to 30-month-olds. BPS Cognit Sect Bull. 2019;5. 10.1111/infa.12431 34418253

[42] Hendry A, Johnson MH, Holmboe K. Early development of visual attention: change, stability, and longitudinal associations. Ann Rev Dev Psychol. 2019;1(1).

[43] Hendry A, Jones EJH, Charman T. Executive function in the first three years of life: precursors, predictors and patterns. Dev Rev. 2016;42:1–33. 10.1016/j.dr.2016.06.005.

[44] Hendry A, Jones EJH, Andersson-Konke L, Agyapong M, Bazelmans T, Begum-Ali J, Ersoy M, Goodwin A, Pasco G, Falck-Ytter T, Johnson MH, Charman T, the EASE and STAARS Teams. Family history of ADHD associates with stronger problem-solving skills amongst 2- to 3-year-olds. JCPP Adv. 2015 10.1002/jcv2.70009 PMC12698283 41395339

[45] Hendry A, Scerif G. Moulding environmental contexts to optimise neurodiverse executive function performance and development: a goodness-of-fit account. Infant and Child Development; 2023

[46] Hendry A, Nosyk M, Hulks V, Hudson J, Constable L, Charman T, Mathers S, Rhodes S, Scerif G. Protocol for a feasibility Randomised Control Trial of the Supporting Toddlers with a connection to autism or ADHD to develop strong Attention, Regulation and Thinking skills (START) programme. Pilot Feasibility Stud. 2025 10.1186/s40814-025-01697-3 PMC12492924 41039569

[47] Hulks V, Scerif G, Rhodes S, Smith S, Charman T, Mathers S, Hendry A. Feasibility and acceptability of a parent‐toddler programme to support the development of executive functions in children at elevated likelihood of autism or ADHD: Pilot findings. J Res Spec Educ Needs. 2024;

[48] Hutchison SM, Müller U, Iarocci G. Parent Reports of Executive Function Associated with Functional Communication and Conversational Skills Among School Age Children With and Without Autism Spectrum Disorder. J Autism Dev Disord. 2020;50(6):2019–29. 10.1007/s10803-019-03958-6. 30847709 10.1007/s10803-019-03958-6

[49] Jeong J, Franchett EE, Ramos de Oliveira CV, Rehmani K, Yousafzai AK. Parenting interventions to promote early child development in the first three years of life: a global systematic review and meta-analysis. PLoS Med. 2021;18(5): e1003602. 33970913 10.1371/journal.pmed.1003602 PMC8109838

[50] Kanevski M, Booth JN, Oldridge J, McDougal E, Stewart TM, McGeown S, Rhodes SM. The relationship between cognition and mathematics in children with attention-deficit/hyperactivity disorder: a systematic review. Child Neuropsychol. 2022;28(3):394–426. 34724883 10.1080/09297049.2021.1985444

[51] Kapp SK, Gillespie-Lynch K, Sherman LE, Hutman T. Deficit, difference, or both? Autism and neurodiversity. Dev Psychol. 2013;49(1):59. 22545843 10.1037/a0028353

[52] Kassai R, Futo J, Demetrovics Z, Takacs ZK. A meta-analysis of the experimental evidence on the near-and far-transfer effects among children’s executive function skills. Psychol Bull. 2019;145(2):165. 30652908 10.1037/bul0000180

[53] Kwan S, Jordao J, Spoelstra M, Antflick J, Southward C, Andrade B, Lynch S, Mitchell J, Nicolson R, Iaboni A, Cowan K, Anagnostou E. Community Priorities for Research on Neurodevelopmental Disorders;2018

[54] Lai CLE, Lau Z, Lui SS, Lok E, Tam V, Chan Q, Cheng KM, Lam SM, Cheung EF. Meta-analysis of neuropsychological measures of executive functioning in children and adolescents with high-functioning autism spectrum disorder. Autism Res. 2017;10(5):911–39. 27874266 10.1002/aur.1723

[55] Lawson GM, Hook CJ, Farah MJ. A meta-analysis of the relationship between socioeconomic status and executive function performance among children. Dev Sci. 2018;21(2): e12529. 10.1111/desc.12529 PMC5821589 28557154

[56] Lawson RA, Papadakis AA, Higginson CI, Barnett JE, Wills MC, Strang JF, Wallace GL, Kenworthy L. Everyday executive function impairments predict comorbid psychopathology in autism spectrum and attention deficit hyperactivity disorders. Neuropsychology. 2015;29(3):445. 25313979 10.1037/neu0000145

[57] Leadbitter K, Buckle KL, Ellis C, Dekker M. Autistic self-advocacy and the neurodiversity movement: Implications for autism early intervention research and practice. Front Psychol. 2021;782. 10.3389/fpsyg.2021.635690 PMC8075160 33912110

[58] Lim AK, Rhodes S, Cowan K, O’Hare A. Joint production of research priorities to improve the lives of those with childhood onset conditions that impair learning: the James Lind Alliance Priority Setting Partnership for ‘learning difficulties.’ BMJ Open. 2019;9(10): e028780. 31672710 10.1136/bmjopen-2018-028780 PMC6832015

[59] Masterson D, Lindenfalk B, Kjellström S, Robert G, Ockander M. Mechanisms for co-designing and co-producing health and social care: a realist synthesis. Res Involv Engag. 2024;10(1):103. 10.1186/s40900-024-00638-3 PMC11468303 39390518

[60] McAllister S, Simpson A, Tsianakas V, Canham N, De Meo V, Stone C, Robert G. Developing a theory-informed complex intervention to improve nurse–patient therapeutic engagement employing experience-based co-design and the behaviour change wheel: an acute mental health ward case study. BMJ Open. 2021;11(5): e047114. 33986066 10.1136/bmjopen-2020-047114 PMC8126294

[61] McDougal E, Gracie H, Oldridge J, Stewart TM, Booth JN, Rhodes SM. Relationships between cognition and literacy in children with attention-deficit/hyperactivity disorder: a systematic review and meta-analysis. Br J Dev Psychol. 2022;40(1):130–50. 34605577 10.1111/bjdp.12395 PMC9292415

[62] McLean RL, Harrison AJ, Zimak E, Joseph RM, Morrow EM. Executive function in probands with autism with average IQ and their unaffected first-degree relatives. J Am Acad Child Adolesc Psychiatry. 2014;53(9):1001–9. 10.1016/j.jaac.2014.05.019. 25151423 10.1016/j.jaac.2014.05.019 PMC4144046

[63] Miller M, Arnett AB, Shephard E, Charman T, Gustafsson HC, Joseph HM, Karalunas S, Nigg JT, Polanczyk GV, Sullivan EL, Jones EJH. Delineating early developmental pathways to ADHD: setting an international research agenda. JCPP Adv. 2023;3(2): e12144. 10.1002/jcv2.12144. 37753147 10.1002/jcv2.12144 PMC10519745

[64] Miller S, Dunne L. Peep learning together programme evaluation report; 2020

[65] Miller M, Musser ED, Young GS, Olson B, Steiner RD, Nigg JT. Sibling recurrence risk and crossaggregation of attention-deficit/hyperactivity disorder and autism spectrum disorder. JAMA Pediatr. 2019;173(2):147–52. 30535156 10.1001/jamapediatrics.2018.4076 PMC6439602

[66] Morris C, Simkiss D, Busk M, Morris M, Allard A, Denness J, Janssens A, Stimson A, Coghill J, Robinson K. Setting research priorities to improve the health of children and young people with neurodisability: a British Academy of Childhood Disability-James Lind Alliance Research Priority Setting Partnership. BMJ Open. 2015;5(1): e006233. 25631309 10.1136/bmjopen-2014-006233 PMC4316435

[67] Nicolaidis C, Raymaker D, Kapp SK, Baggs A, Ashkenazy E, McDonald K, Weiner M, Maslak J, Hunter M, Joyce A. The AASPIRE practice-based guidelines for the inclusion of autistic adults in research as co-researchers and study participants. Autism. 2019;23(8):2007–19. 30939892 10.1177/1362361319830523 PMC6776684

[68] Nosyk M, Hendry A. Toddlers (but not infants) with elevated autistic traits show lower executive function scores. INSAR 2023;2023

[69] O’Reilly F, Scerif G. Enhancing preschool executive function: a systematic review and meta-analysis of parent-led interventions. OSF;2024

[70] Oakes LM, Rakison DH. Developmental cascades: Building the infant mind. Oxford: Oxford University Press; 2019.

[71] Olde Dubbelink LM, Geurts HM. Planning skills in autism spectrum disorder across the lifespan: a meta-analysis and meta-regression. J Autism Dev Disord. 2017;47(4):1148–65. 28160225 10.1007/s10803-016-3013-0 PMC5357294

[72] Peeple. Learning Together Programme: Programme Content;2023. Retrieved 20/06/2023 from https://www.peeple.org.uk/ltp-content

[73] Prime H, Andrews K, Markwell A, Gonzalez A, Janus M, Tricco AC, Bennett T, Atkinson L. Positive parenting and early childhood cognition: a systematic review and meta-analysis of randomized controlled trials. Clin Child Fam Psychol Rev. 2023;26(2):362–400. 10.1007/s10567-022-00423-2. 36729307 10.1007/s10567-022-00423-2 PMC10123053

[74] Robson DA, Allen MS, Howard SJ. Self-regulation in childhood as a predictor of future outcomes: a meta-analytic review. Psychol Bull. 2020;146(4):324. 31904248 10.1037/bul0000227

[75] Roebers CM. Executive function and metacognition: towards a unifying framework of cognitive self-regulation. Dev Rev. 2017;45:31–51. 10.1016/j.dr.2017.04.001.

[76] Rommelse NNJ, Altink ME, Oosterlaan J, Buschgens CJM, Buitelaar J, Sergeant JA. Support for an independent familial segregation of executive and intelligence endophenotypes in ADHD families. Psychol Med. 2008;38(11):1595–606. 10.1017/S0033291708002869. 18261248 10.1017/S0033291708002869

[77] Rommelse NNJ, Geurts HM, Franke B, Buitelaar JK, Hartman CA. A review on cognitive and brain endophenotypes that may be common in autism spectrum disorder and attention-deficit/hyperactivity disorder and facilitate the search for pleiotropic genes. Neurosci Biobehav Rev. 2011;35(6):1363–96. 10.1016/j.neubiorev.2011.02.015. 21382410 10.1016/j.neubiorev.2011.02.015

[78] Russell G, Rodgers LR, Ukoumunne OC, Ford T. Prevalence of parent-reported ASD and ADHD in the UK: findings from the Millennium Cohort Study. J Autism Dev Disord. 2014;44:31–40. 23719853 10.1007/s10803-013-1849-0

[79] Sapiets S. Methods for co-production in research and practice. Bild International PBS Conference, Bristol; 2022

[80] Scheerer NE, Pourtousi A, Yang C, Ding Z, Stojanoski B, Anagnostou E, Nicolson R, Kelley E, Georgiades S, Crosbie J, Schachar R, Ayub M, Stevenson RA. Transdiagnostic patterns of sensory processing in autism and ADHD. J Autism Dev Disord. 2022. 10.1007/s10803-022-05798-3. 36306002 10.1007/s10803-022-05798-3

[81] Schuck R, Lin F, Geng A, Doss Y, Crousure H, Dwyer P, Baiden KMP, Williams ZJ, Wang M. A Qualitative Inquiry into Autistic Adult’s Views on Intervention Goals for Young Autistic Children. Stockholm: INSAR; 2023.

[82] Semenov AD, Zelazo PD. Mindful family routines and the cultivation of executive function skills in childhood. Hum Dev. 2019;63(2):112–31.

[83] Seng G-J, Tseng W-L, Chiu Y-N, Tsai W-C, Wu Y-Y, Gau SS-F. Executive functions in youths with autism spectrum disorder and their unaffected siblings. Psychol Med. 2021;51(15):2571–80. 10.1017/S0033291720001075. 32349803 10.1017/S0033291720001075

[84] Sjöwall D, Thorell LB. Neuropsychological deficits in relation to ADHD symptoms, quality of life, and daily life functioning in young adulthood. Appl Neuropsychol Adult. 2022;29(1):32–40. 10.1080/23279095.2019.1704287. 31881160 10.1080/23279095.2019.1704287

[85] Skivington K, Matthews L, Simpson SA, Craig P, Baird J, Blazeby JM, Boyd KA, Craig N, French DP, McIntosh E. A new framework for developing and evaluating complex interventions: update of Medical Research Council guidance. Bmj. 2021;374. 10.1136/bmj.n2061 PMC8482308 34593508

[86] Snyder HR, Miyake A, Hankin BL. Advancing understanding of executive function impairments and psychopathology: bridging the gap between clinical and cognitive approaches. Front Psychol. 2015;6:328. 10.3389/fpsyg.2015.00728. 25859234 10.3389/fpsyg.2015.00328 PMC4374537

[87] St John T, Estes AM, Hazlett HC, Marrus N, Burrows CA, Donovan K, Torres Gomez S, Grzadzinski RL, Parish-Morris J, Smith R, Styner M, Garic D, Pandey J, Lee CM, Schultz RT, Botteron KN, Zwaigenbaum L, Piven J, Dager SR, Network I. Association of sex with neurobehavioral markers of executive function in 2-year-olds at high and low likelihood of autism. JAMA Netw Open. 2023;6(5):e2311543–e2311543. 10.1001/jamanetworkopen.2023.11543. 37140923 10.1001/jamanetworkopen.2023.11543 PMC10160873

[88] Staniszewska S, Brett J, Simera I, Seers K, Mockford C, Goodlad S, Altman D, Moher D, Barber R, Denegri S. GRIPP2 reporting checklists: tools to improve reporting of patient and public involvement in research. Bmj. 2017;358. 10.1136/bmj.j3453 PMC5539518 28768629

[89] Stern A, Pollak Y, Bonne O, Malik E, Maeir A. The relationship between executive functions and quality of life in adults with ADHD. J Atten Disord. 2017;21(4):323–30. 24189201 10.1177/1087054713504133

[90] Teepe RC, Molenaar I, Oostdam R, Fukkink R, Verhoeven L. Children’s executive and social functioning and family context as predictors of preschool vocabulary. Learn Individual Differ. 2017;57:1–8. 10.1016/j.lindif.2017.05.012.

[91] Valcan DS, Davis H, Pino-Pasternak D. Parental behaviours predicting early childhood executive functions: a meta-analysis. Educ Psychol Rev. 2018;30(3):607–49.

[92] Van Eylen L, Boets B, Cosemans N, Peeters H, Steyaert J, Wagemans J, Noens I. Executive functioning and local-global visual processing: candidate endophenotypes for autism spectrum disorder? J Child Psychol Psychiatry. 2017;58(3):258–69. 10.1111/jcpp.12637. 27804132 10.1111/jcpp.12637

[93] Wallace GL, Kenworthy L, Pugliese CE, Popal HS, White EI, Brodsky E, Martin A. Real-world executive functions in adults with autism spectrum disorder: profiles of impairment and associations with adaptive functioning and co-morbid anxiety and depression. J Autism Dev Disord. 2016;46(3):1071–83. 10.1007/s10803-015-2655-7. 26572659 10.1007/s10803-015-2655-7 PMC5111802

[94] Wallace GL, Yerys BE, Peng C, Dlugi E, Anthony LG, Kenworthy L. Assessment and treatment of executive function impairments in autism spectrum disorder: an update. Int Rev Res Dev Disabil. 2016;51:85–122.

[95] Wass SV. Applying cognitive training to target executive functions during early development. Child Neuropsychol. 2015;21(2):150–66. 10.1080/09297049.2014.882888. 24511910 10.1080/09297049.2014.882888 PMC4270409

[96] Willcutt EG, Doyle AE, Nigg JT, Faraone SV, Pennington BF. Validity of the executive function theory of attention-deficit/hyperactivity disorder: a meta-analytic review. Biol Psychiat. 2005;57(11):1336–46. 10.1016/j.biopsych.2005.02.006. 15950006 10.1016/j.biopsych.2005.02.006

[97] Zelazo PD. Executive function: reflection, iterative reprocessing, complexity, and the developing brain. Dev Rev. 2015;38:55–68. 10.1016/j.dr.2015.07.001.

