# Supplementary material for: Learning from the community: iterative co-production of a programme to support the development of attention, regulation and thinking skills in toddlers at elevated likelihood of autism or ADHD
Source: Res Involv Engagem. 2025 Jan 24;11:7. doi: 10.1186/s40900-025-00674-7 (PMC11762902; doi:10.1186/s40900-025-00674-7)
Supplement: Supplementary file 1 — Additional file 1. [file 40900_2025_674_MOESM1_ESM.docx]

# Appendix 1: GUIDED checklist

| **Item description** | **Manuscript page** |
| --- | --- |
| 1. Report the context for which the intervention was developed. | 3-5 |
| 2. Report the purpose of the intervention development process. | 7-9 |
| 3. Report the target population for the intervention development process. | 5-6, 27 |
| 4. Report how any published intervention development approach contributed to the development process | 9 |
| 5. Report how evidence from different sources informed the intervention development process. | 3-5, 10 |
| 6. Report how/if published theory informed the intervention development process. | 3-5, 21 |
| 7. Report any use of components from an existing intervention in the current intervention development process. | 7, 12 |
| 8. Report any guiding principles, people or factors that were prioritised when making decisions during the intervention development process. | 5-7 |
| 9. Report how stakeholders contributed to the intervention development process. | 10-16 |
| 10. Report how the intervention changed in content and format from the start of the intervention development process. | 23-30 |
| 11. Report any changes to interventions required or likely to be required for subgroups | N/A |
| 12. Report important uncertainties at the end of the intervention development process. | 35-36 |
| 13. Follow TIDieR guidance when describing the developed intervention. | See accompanying protocol paper |
| 14. Report the intervention development process in an open access format. | Submitted to an Open Access journal |

# Appendix 2: GRIPP2-SF checklist

| **Section and topic** | **Item** | **Reported on page No** |
| --- | --- | --- |
| 1: Aim | Report the aim of PPI in the study | 9, 11-15 |
| 2: Methods | Provide a clear description of the methods used for PPI in the study | 9-16 |
| 3: Study results | Outcomes—Report the results of PPI in the study, including both positive and negative outcomes | 17-37 |
| 4: Discussion and conclusions | Outcomes—Comment on the extent to which PPI influenced the study overall. Describe positive and negative effects | 33-37 |
| 5: Reflections/ critical perspective | Comment critically on the study, reflecting on the things that went well and those that did not, so others can learn from this experience | 33-36 |

# Appendix 3a: Profile of families attending the proof of concept sessions

Participants in this initial stage were not screened in terms of their family history of autism or ADHD, but recent research indicates that, in the UK, family history of autism and ADHD is highest within socio-economically disadvantaged communities (Bazelmans, Scerif, Holmboe, Gonzalez-Gomez, & Hendry, 2023). In most cases, families comprised mother-and-child, but for two families the parent attending was the father, and in three instances a grandmother came along with (or sometimes instead of) the parent. In instances where the grandmother attended in lieu of the parent, their feedback was collected and is summarised with the parent feedback. Two other families occasionally brought other family members (the child’s aunt) and at one session a key worker assisting the child’s parent attended; these individuals were not asked to complete the feedback questionnaire. The children attending the sessions were aged 7- to 19 months; this age range was deliberately broad to reflect the likely range of developmental ability that might be found in a neurodiverse group of toddlers. It was observed that families were from a range of ethnic backgrounds.

# Appendix 3b: Parent questionnaire at proof of concept stage

The parent questionnaire was administered via a postcard handed out to each parent at the end of each session, which they completed anonymously and then posted into a sealed box. The parent questionnaire featuring the following questions:

- How often do you already do the kinds of activities described in this session with your child? (Never (1) / Not very often (2) / Sometimes (3) / Often (4))
- How would you rate your confidence about this topic after the session? (Not at all confident (1) / Only a little confident (2) / Quite confident (3) / Very confident (4))
- How likely are you to do the kinds of activities described in this session with your child over the next few weeks? (Not at all likely (1) / Not very likely (2) / Quite likely (3) / Very likely (4))
- Please add any comments about things you found useful or confusing (but don’t provide any identifying information like names).

# Appendix 4: Content refinement workshops agenda and protocol

On expression of interest, potential panel members were asked to summarise their connection to autism/ADHD, their child(ren)’s age in years, if they had any specific access needs (all participants were told that core material would be shared beforehand for people who prefer to have time to read and reflect, but that there was no expectation to do any preparation work), and identify their preferred day and time for the online workshops.

Agenda for Workshop 1a and Workshop 1b (panel members attended only 1 of these)

- Introductions and ground rules
- Overview of the project rationale and aims
- Comments on the structure of the programme, and the delivery and evaluation approach
- In-depth discussion of Session 1, 2 and 3 materials

Agenda for Workshop 2

- In-depth review of Session 4, 5 and 7 materials (session 6 is a recap session)

Agenda for Workshop 3

- In-depth review of Session 8, 9 and 10 materials (session 12 is a recap session, and session 11 is an extension of previous weeks)

Each workshop lasted 2.5 hours (including breaks). Session handouts were sent in advance, and shared online during the session, whilst the session plans were disaggregated into sub-sections for review via an online discussion tool (Padlet). For each sub-section of the session plan, panel members were asked to indicate how positively they felt about the topic and proposed content, on a scale of 1 (very negative) to 5 (very positive). Low scores were prioritised for discussion, but additionally panel members could add comments to every section. Members were invited to comment on ways in which the content could be improved (to improve inclusivity and alignment to a neurodiversity-affirming approach, or parent and child engagement more generally), and provide examples or tips from their own experience. Comments were presented anonymously but panel members were invited to elaborate verbally or through the chat function if they wished. During the workshops, panel members were given the option to participate with their camera on or off, using spoken or written inputs as preferred. Spoken discussion was recorded and transcribed to help with note taking. Written contributions during the Padlet portion of the workshops were exported. During these workshops the programme logic model and delivery and evaluation approach was also interrogated and refined.

# Appendix 5: Detailed review of outputs from prior consultation exercises

*Supplementary Table 5.1*. Summary of James Lind Alliance (JLA) Priority Setting Partnerships (PSP) priorities relevant to the proposal

| PSP source | Priority | Relevance to programme?^a^ |
| --- | --- | --- |
| Autism | Which interventions improve mental health or reduce mental health problems in autistic people? How should mental health interventions be adapted for the needs of autistic people? | Yes |
|  | Which interventions are effective in the development of communication/language skills in autism? | Yes |
|  | What are the most effective ways to support/provide social care for autistic adults? | No |
|  | Which interventions reduce anxiety in autistic people? | Yes |
|  | Which environments/supports are most appropriate in terms of achieving the best education/ life/ social skills outcomes in autistic people? | Yes |
|  | How can parents and family members be supported/educated to care for and better understand an autistic relative? | Yes |
|  | How can autism diagnostic criteria be made more relevant for the adult population? And how do we ensure that autistic adults are appropriately diagnosed? | No |
|  | How can we encourage employers to apply person-centred interventions & support to help autistic people maximise their potential and performance in the workplace? | No |
|  | How can sensory processing in autism be better understood? | Yes |
|  | How should service delivery for autistic people be improved and adapted in order to meet their needs? | No |
| Childhood Disability | Does the timing and intensity of therapies (e.g. physical, occupational and speech and language therapy, ‘early intervention’, providing information etc.) alter the effectiveness of therapies for infants and young children with neurodisability, including those without specific diagnosis? What is the appropriate age of onset / strategies / dosage / direction of therapy interventions? | Yes |
|  | To improve communication for children and young people with neurodisability: (a) what is the best way to select the most appropriate communication strategies? And (b) how to encourage staff/carers to use these strategies to enable communication? | Yes |
|  | Are child-centred strategies to improve children’s (i.e. peers) attitudes towards disability (e.g. buddy or Circle of Friends etc) effective to improve inclusion and participation within educational, social and community settings? | No |
|  | Does appropriate provision of wheelchairs to enable independent mobility for very young children improve their self-efficacy? | No |
|  | Are counselling/psychological strategies (e.g. talking therapies) effective to promote the mental health of children and young people with neurodisability? | No |
|  | What is the (long term) comparative safety and effectiveness of medical and surgical spasticity management techniques (Botulinum neurotoxin A (BoNT-A), Selective Dorsal Rhizotomy (SDR), Intrathecal Baclofen (ITB), orally administered medicines) in children and young people with neurodisability? | No |
|  | Does a structured training programme, medicines and/or surgery speed up the achievement of continence (either/or faecal or urinary) for children and young people with neurodisability? | No |
|  | What strategies are effective to improve engagement in physical activity (to improve fitness, reduce obesity etc.) for children and young people with neurodisability? | No |
|  | Which school characteristics (e.g. policies, attitudes of staff etc.) are most effective to promote inclusion of children and young people with neurodisability in education and after-school clubs? | No |
|  | What is the long term safety, effectiveness and sustainability of behavioural strategies and/or drugs (e.g. melatonin) to manage sleep disturbance in children and young people with neurodisability (outcomes include time to onset, duration, and reducing impact on family)? | No |
| Learning Difficulties (Scotland) | What knowledge, skills and training do educational professionals need to identify the early signs of learning difficulties and provide optimal support for children and young people affected to help them achieve the best possible outcomes? | No |
|  | What is the best educational and community environment for children and young people with learning difficulties? | Yes |
|  | How can multiple types of professionals work together with parents and carers to improve identification, diagnosis, interventions and treatments and achieve the best outcomes for children and young people with learning difficulties? | No |
|  | Which early interventions are effective for children and young people with learning difficulties, at what ages and stages are they best introduced and what are the long-term outcomes? | Yes |
|  | What knowledge, skills and training do health, social work and “third sector” (e.g. charities and support services) professionals need to understand the best support to give children and young people with learning difficulties and their families/carers? | No |
|  | How can parents, carers, brothers and sisters and extended families of children and young people with learning difficulties, be best supported to achieve their best quality of life before, during and after the diagnosis or identification in home, school and community contexts? | Yes |
|  | How can we best identify early features, symptoms and signs of learning difficulties amongst children, young people and their families/carers? | Yes |
|  | What is the best way to assess learning difficulties in children and young people? | Yes |
|  | Which strategies are effective in preventing stigma and bullying towards children and young people with learning difficulties? | No |
|  | Which strategies are effective in helping children and young people with learning difficulties live independent lives, including during times of transitions? | Yes |
| Neuro-developmental Disorders (Canada) | What are the most effective treatment options/plans (e.g., timing, frequency, duration, type, intensity or dosage) for individuals with neurodevelopmental disorders for both short and long-term benefits? | Yes |
|  | How can system navigation be organized in a manner that enables coordinated services and supports across the lifespan for individuals with neurodevelopmental disorders and their families? | No |
|  | Which biological treatments (including medications, gene therapy, stem cell therapy, etc.) are effective for neurodevelopmental disorders and associated symptoms? | No |
|  | Which child and family-centered interventions or approaches promote optimal individual and family functioning? | Yes |
|  | Which interventions best help individuals with neurodevelopmental disorders develop emotional and behavioural regulation (including increasing impulse control and reducing compulsive behaviour)? | Yes |
|  | Which resources are needed to more effectively address the health, social and emotional needs of families or caregivers of individuals with neurodevelopmental disorders? | No |
|  | How can treatment decisions for individuals with neurodevelopmental disorders be more precise (i.e., based on the diagnosis, age, functional need of the individual)? | No |
|  | Which are the most effective pharmacological and non-pharmacological treatments for aggressive and self-injurious behaviour in individuals with neurodevelopmental disorders? | Yes. |
|  | Which are the most effective pharmacological and non-pharmacological intervention(s) to reduce anxiety in individuals with neurodevelopmental disorders? | Yes |
|  | Which interventions are most effective to help individuals with neurodevelopmental disorders improve their social skills and develop and maintain social relationships? | Yes |

^a^ If Yes, the specific relevance is detailed in Table 1 in the main manuscript.

# Appendix 6: Draft programme logic model

# Appendix 7: Detail of consultation within community stakeholders to check acceptability of the programme and evaluation proposal.

*Supplementary Table 6.1.* Responses on the programme and evaluation proposal from 2 neurodivergent parents and a broader panel of neurodivergent adults

|  | Areas of strength | Areas to strengthen or change | Changes made |
| --- | --- | --- | --- |
| Reviewer 1 | a) My initial thoughts on the study are that it has great potential for improving the outcomes for all those involved (which is always a good thing!).  b) Definitely highlight the potential for improvement to the health and wellbeing of those with EF difficulties, and the practical nature of the approach. This is about learning practical skills for autistic parents as well as potentially autistic children (though I understand their youth and elasticity is the reason they are being used, but learning strategies is always helpful). Autistica’s survey into what autistic adults want research to focus on https://www.autistica.org.uk/our-research/our-research/your-research-priorities - number 1 is “Which interventions improve mental health or reduce mental health problems in autistic people?” and I think this sort of thing is relevant.  c) Make sure the inclusion of autistic collaboration and advice is stressed | i) reduce the use of ‘with autism’/’pathway to autism’ and other potentially problematic language that can be considered either too ambiguous or ‘othering’.  ii) consider whether peer support needs to be in person  iii) I would prefer to prioritise autistic parents in the study, due to offering additional advantages to the autism community by building peer support networks for autistic parents, and through the benefit of EF strategies for parents who may need them more than the average parent. | i) Identity-first language used by default when discussing autistic people.  ii) The question of whether the groups should be in person or online deferred to the next stage of development.  iii) Recruitment strategy updated to include autistic parent networks. The peer support aspect of the programme given more emphasis. |
| Reviewer 2 | a) I think executive functioning is something that most autistic people would like to improve  b) Session reminders are good because there is a high chance people with EF problems might not turn up because they forget or get the time wrong (speaking from experience)  c) It is good that you are doing both verbal and written summaries of the missed sessions to make it more accessible.  d) Highlight the importance of not seeking to ‘normalise’ autistic children through the programme (as this is linked to masking, and in turn to elevated suicide risk): mental health isn’t usually considered when assessing the success of an intervention but it really should be. | i) Use identity first language where possible, particularly re autism  ii) Record the sessions with audio only (not video) as many autistic people hate being videoed  iii) Need to check that parents are interpreting the parent-report questionnaires as intended  iv) Add detail on how you will make the sessions as accessible as possible. E.g. consider the sensory environment, allow parents to see the room in advance, give clear directions with pictures, meet people at the door of the building rather than expecting them to get to the room on their own. Show people out to reception afterwards if they want that.  v) Include self-identification as acceptable for inclusion because there are many barriers to diagnosis.  vi) Be particularly careful with discussion of parent-child relationships due to harmful and inaccurate stereotypes around neurodivergent parents, and ensure that sessions do not parent blame and that non-attendance is not presented as poor parenting. | i) Identity-first language used by default when discussing autistic people.  ii) Sessions to be audio recorded not video recorded.  iii) Questionnaires to be piloted with autistic parents in the next phase.  iv) Venue accessibility reviews added to budget and schedule. Welcome pack to include venue photos and maps. Practitioner training to include inclusivity and accessibility training.  v) Self-identification, with high scores on relevant trait questionnaire, added as a possible inclusion criterion  vi) Recruitment and referral materials, information sheets and programme materials to be checked for non-stigmatizing /blaming language. |
| Autistica panel | a) EF is something autistic people and families want help with  b) RCTs are too rare in autism research, early intervention/support is important but under-evidenced; your approach to research is robust c) Your communication about autistic people is sensitive and positive. |  |  |

Comments have been paraphrased where necessary, in the interests of brevity.

# Appendix 9: Advisory Group review

*Supplementary Table 7.1.* Advisory Group comments on the programme materials

| Member | Focus | Comment |
| --- | --- | --- |
| AG1 | Session materials | Overall, I think the programme is brilliant. I loved reading it all. From a teaching perspective the activities were spot on, I just had a few comments and suggestions about content. From a parenting perspective, I would have loved to have done this programme with XXX! There would have been elements of it that he would have found tricky, but it is all easily modifiable and the idea that there is no pressure or judgement is clear. Like you said, there are aspects of it that are similar to what we were taught to do by an SLT. However, making the EF explicit and building all the different aspects of EF feels very important. I am so excited to see the outcome of this. My hunch as a SEND parent and practitioner is that it will have a significant impact. |
| AG3 | Session plan notes: “Aim to have fixed any obvious sensory hazards (e.g. flickering lights, competing patterns on floor /walls) before the session starts but explain to parents that if there’s anything in the space making them uncomfortable they can mention it either now or later in private and you’ll try to solve it. | Just want to say I love this, I think it's great! I wish all courses did this |
| AG3 | Session 1: it is important to recognise and value what families already know and are doing – keeping the ORIM framework in mind will help you to do so | Love this- it is so important in the first session allow the parents to see what they are already doing and feel recognised for this |
| AG3 | Now and next board | I really like this idea and I think it would be incredibly useful in a group session to have this. It would make me feel more relaxed as a participant and I would also know at what point I might have to step away which would help me prepare. |
| AG3 | Session 1: efforts re inclusivity | I hope you recognise that hardly anyone else even considers these aspects when designing a course - you really are creating something fantastic! |
| AG1 | Session 1 | These are great games and are easily adaptable to the child’s current fascinations. |
| AG2 | Session 2 key ideas | I have no changes to suggest to this, I think it’s clear and sympathetically written |
| AG2 | Session 2 session plan | -Sensory overload. Again I like this, especially how you’ve noted that triggers will change in circumstances. I also like how you have addressed stimming, it’s a good balance. |
| AG2 | Session 2 session plan | Experiencing being soothed by a loved one helps toddlers to develop the ability to calm themselves down as they grow up . I love that this has been emphasised, it’s such an important message. As a midwife I saw a fairly common assumption that comfort would ‘spoil’ a baby, even very tiny ones, making them get used to it and expect it (rod for your on back etc). It’s so important to make it clear this is not the case, as you have done here. |
| AG1 | Session 2 session plan: stimming | Love this paragraph – really important to communicate the benefits of stimming |
| AG3 | Session 3: TDAH | I really like the TDAH sheet. I find it very inclusive and the tasks are very simple, plus using household objects everyone will have is great as well. |
| AG3 | Session 3: Session plan | I love that you have included this. This would be my child if there was a car or train- he would want to stay with that and not move on and usually I'd feel excluded from the group but you have made it possible to still feel included by putting this explicitly in the notes |
| AG2 | Session 4: Session plan | I love the point of using screen time thoughtfully. This is way more helpful than screens = bad message, which people just turn off from because screens are part of life, and a coping mechanism for many parents. In fact, I think the way screen use is addressed is helpful and respectful altogether. It would be a very helpful guide for all families. |
| AG2 | Session 4: Session plan | Activity- I like the suggestions for activities, and that children can choose from a good variety. I like the emphasis on the fact that there is no correct way to play and think this would be a really good chance for the two practitioners to support parents by role modelling this. |
| AG6 | Session plans | Overall I think the session plans are great and there has been a lot of thought put into diversity and being flexible. |
| AG5 | Session 4: Session plan, screens | I like the way you’ve sensitively tackled this topic in this section, great suggestions. |
| AG3 | Session 5: Note about some variation in filtering being fixed | I really appreciate the inclusion of this. I can't tell you how many times I have been told it's not that bad just get over it- as if I had any choice in the matter! I think it's very empowering for parents to learn this about their child and/or themselves |
| AG3 | Session 5 activities | I like the three different options provided. At this age my child would not have joined in with a group activity so having the option to do it just with me would have made it possible to participate. |
| AG3 | Session 8 presentation | This would be a great worksheet to use again and again. I think it would really help parents to fully understand all the barriers their child may have in the way and also why thay can control their impluse in one situation but not in another- also I am definitely going to use this idea to help explain some things to family members as to why my child behaves the way he does sometimes so a big thank you for making it a bit easier for me! :) |
| AG3 | Session 10 key ideas (resilience not about ignoring discomfort/distress) | I love that this is included. It's really, really important to get this point across. Too often resilience is framed in this way and it's toxic |
| AG3 | Session 10 session plan | This is such a relatable example! it made me laugh ☺️ I think it will also help parents to recognise when their child is actually really persistent!!! |
| AG3 | Session 10 book suggestion (Umar) | What a lovely book! That's going on my wish list! |
| AG3 | PPI overall | I really appreciated this opportunity to see the revised materials and comment on them once again. It's been really fun and you are creating something amazing! I am looking forward to seeing how it all progresses! |
| AG4 | Session 8 presentation | I did really like the set of slides about goal setting and thought that was such a useful resource to help understand. |
| AG5 | Session 8 presentation and key ideas | I liked everything about the key ideas and activities handouts, so haven’t got any suggestions or feedback for those. I also really like the slides about the goals/barriers/supports.  The star is a lovely image, it’s a great way to illustrate the concepts being discussed. |
| AG5 | Session 8 session plan point about highly controlling parening | So glad you’re highlighting this. A lot of parents of neurodivergent children get told that they need to be stricter (i.e. use more punishments and control). |
| AG5 | Session 8 session plan point about adjusting expectations | I really like the way you’ve acknowledged these differences and how reasonable expectations might differ between children |
| AG5 | Session 8 activities | I think this is a great variety of activity ideas suitable for a wide range of children, my daughter would have love all of these. |
| AG2 | Session 9 session plan | I like the revisiting of stimming, and again the fact that you have highlighted it can be positive |
| AG2 | Session 9 session plan | I think the idea of parents identifying their children’s key issue could work really well. Assuming these are a diverse group of kids, a standard goal could be way out of their reach developmentally, so good to ask where they are at and what matters to the parents. |
| AG2 | Session 9 session plan | Part 2- this section is really spot on, I think it includes different needs nicely. The note about the TADH and imaginary play is good too |
| AG2 | Session 9 session plan | Shape sorter game- I think many kids won’t get this. I think you’ve accommodated for this in how it’s going to be delivered, and it’s good that you’ve once again pointed out that any level of engagement is beneficial |
| AG2 | Session 9 TDAH | How many uses- I think this is a great adaptation to include how autistic people play imaginatively. The creating alternative uses, a more engineering imaginary play than fantasy imaginary play is good- I am sure there are proper words for what I am saying but I hope you understand what I mean. Hopefully it would be accessible to most. |
| AG2 | Session 9 TDAH | What can it be- again I children will be able to engage on different levels. Eg. My lads would have not been able to see blocks as a snake, but they know what a pyramid looks like. |
| AG2 | Session 9 presentation | I like the presentation |
| AG5 | Overall | Being paid to input really makes me feel that you value my time and expertise. That in itself makes more difference than the money. |
| AG7 | Overall | It's been a real pleasure to be involved in the project and I've so appreciated the thoughtful and inclusive approach that you've taken to it. I look forward to seeing how it develops. |
| AG1 | Session 10 session plan (session on the importance of scaffolding to support perseverance) | This is so interesting and from a personal point of view something I wish I had know when XXX was a toddler. I would have worked as hard on this (and other EF skills) as I did on his language. When XXX found activities challenging, he would become very frustrated, and he often became so wound up it turned into a major meltdown. This meant I would often avoid these situations by distracting him and getting him past the difficulty myself, or by abandoning the activity all together. I didn’t have the knowledge or brain space to stick with his frustration and support him with it. I was so keen to avoid meltdowns that I didn’t dare get close to the point of frustration out of fear. Now as an 18 year old he struggles to cope with challenges. He calls him self ‘stupid’ when he can’t do something. He also struggles to shift the belief that people are born knowing how to do something perfectly. He finds the idea of mistakes and building skills and knowledge over time very difficult. |
