# Supplementary figures and images for: Learning from the community: iterative co-production of a programme to support the development of attention, regulation and thinking skills in toddlers at elevated likelihood of autism or ADHD
Source: Res Involv Engagem. 2025 Jan 24;11:7. doi: 10.1186/s40900-025-00674-7 (PMC11762902; doi:10.1186/s40900-025-00674-7)

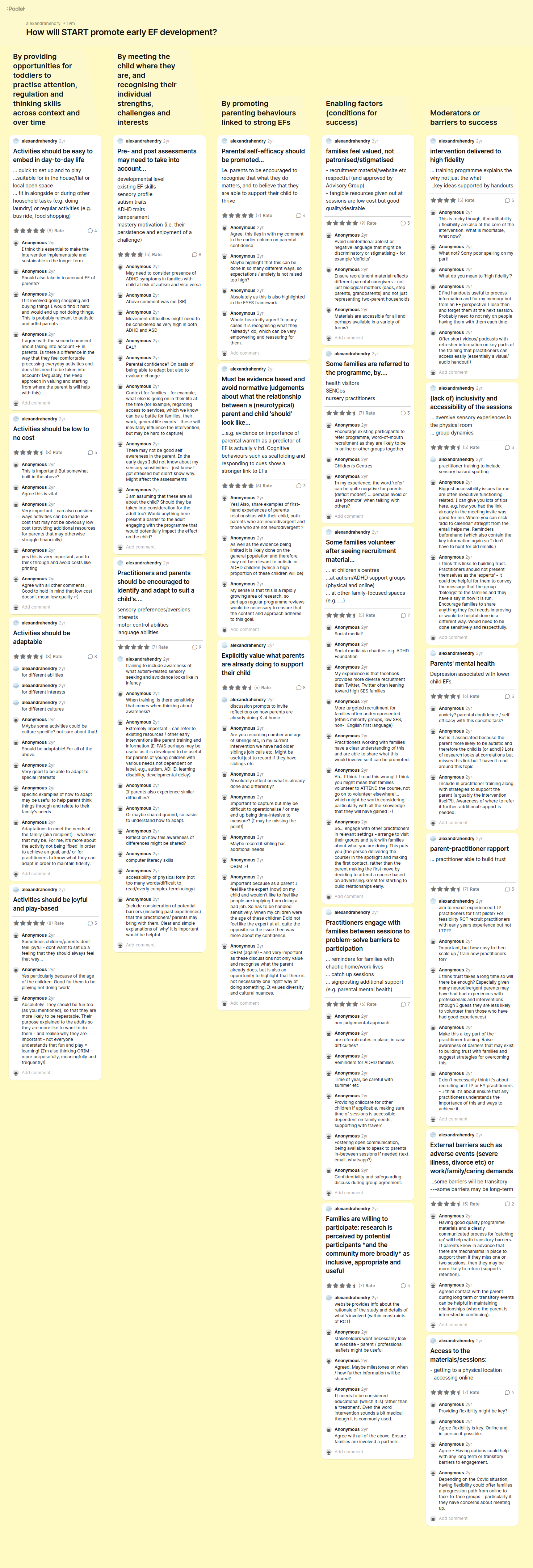

Supplement: Supplementary file 2 — Additional file 2. [file 40900_2025_674_MOESM2_ESM.png]
